# Expression of a modified *Avr3a* gene under the control of a synthetic pathogen‐inducible promoter leads to *Phytophthora infestans* resistance in potato

**DOI:** 10.1111/pbi.14615

**Published:** 2025-03-09

**Authors:** Friedrich Kauder, Gabor Gyetvai, Klaus Schmidt, Daniel Stirnweis, Tobias Haehre, Kai Prenzler, Anja Maeser, Christine Klapprodt, Florian Tiller, Jens Lübeck, Dietmar J. Stahl

**Affiliations:** ^1^ Solana Research GmbH Windeby Germany; ^2^ KWS SAAT SE & Co. KGaA Einbeck Germany; ^3^ Deutsche Saatveredelung AG Asendorf Germany; ^4^ Grillido GmbH Munich Germany

**Keywords:** *Phytophthora infestans*, late blight resistance, potato, synthetic promoter, *Avr3a* avirulence gene, *R3a* resistance gene

## Abstract

Late blight resistance of potato was improved by the co‐expression of the potato resistance gene *R3a* and the pathogen‐inducible avirulence gene *Avr3a* of *Phytopthora infestans*. The synthetic pathogen‐inducible promoter 2xS‐4xD‐NpCABE_core_, which is composed of the *cis*‐acting elements S and D and the core promoter of the *NpCABE* gene, was developed for potato. By analysis of 20 core promoters from Solanacea species synthetic promoters of the 2xS‐2xD‐type were generated which differ in their background activity, strength and promoter inducibility. These data showed that the core promoter plays an important role for the architecture of a synthetic promoter and influences the specificity and strength beside the *cis*‐acting element. The 2xS‐2xD‐NpCABE_core_ promoter was further improved by increasing the number of the *cis*‐acting elements resulting in the 2xS‐4xD‐NpCABE_core_ promoter. Modified *Avr3a* alleles, which triggered less cell death than the *Avr3a* ^KI^ allele, were expressed with the optimized synthetic promoter in transgenic potatoes with an *R3a* gene. The transgenic lines showed less late blight symptoms and up to 60% reduction of sporangia in detached leaf assays. The absence of a negative plant phenotype in the greenhouse demonstrated that the balanced co‐expression of a modified *Avr3a* gene under the control of an optimized synthetic promoter is a promising strategy to increase late blight resistance of potatoes. This concept might be as well applied to other crops since the co‐expression of the *R3a* and *Avr3a* ^*KI*^ gene induced cell death in leaves of corn, wheat and soybean in a transient assay.

## Introduction

Potato is grown in almost all countries of the world. The annual global potato production was 388.7 million metric tons in 2021 (FAO, 2021). After wheat and rice, it is the third crop in order of importance consumed by humans (Haverkort *et al*., 2009). The potato consumption was on average 35 kg per human being in 2013 with large regional differences (Wijesinha‐Bettoni and Mouillé, 2019). The contribution of potatoes to the global food supply is increasing as the consumption more than doubled in developing countries between 1960 and 2005 (Jennings *et al*., 2020). With respect to climate change, it was calculated that potato could benefit from the changing environmental conditions and the potato yields are likely to increase from 9% to 20% globally by 2050 due to CO_2_ and adaptation benefits (Jennings *et al*., 2020). Although the impacts of climate change on potato are favourable compared with other major crops like for corn, rice and wheat (Challinor *et al*., 2014), potato yield and quality are still threatened by diseases.

Late blight caused by the oomycete *Phytophthora infestans* is a major constraint to the production of potato, resulting in lower yield, and tuber rot during storage (Kamoun *et al*., 2015). The most popular control strategy against late blight is complete disease prevention by weekly spraying of a mixture of preventive and systemic fungicides (Ivanov *et al*., 2021). Plant breeding efforts have been made for more than 130 years but have yet to provide a durable solution for late blight resistance (Kamoun *et al*., 2015). *P. infestans* is a diploid organism with a high genetic variation and a high number of effector proteins located in dynamic regions of the genome which might promote rapid adaption to resistant plants. Introgression of single late blight resistance (*Rpi*) genes from the wild species *Solanum demissum* by breeding was not sustainable since the resistance was always broken down (Haverkort *et al*., 2009). More than 20 *Rpi*‐genes have been mapped and cloned from different Solanum species (Witek *et al*., 2021). Stacking several R‐genes selected from *Solanum americanum* (Witek *et al*., 2016; Witek *et al*., 2021), *Solanum bulbocastanum* (Park *et al*., 2005; van der Vossen *et al*., 2005), *Solanum demissum* (Ballvora *et al*., 2002; Huang *et al*., 2005; Vossen *et al*., 2016), *Solanum papita*, *Solanum stoloniferum* and *Solanum venturii* (Foster *et al*., 2009; Pel *et al*., 2009) in potato might be a promising way to develop durable resistance (Haverkort *et al*., 2009; Jo *et al*., 2014; Witek *et al*., 2021).

Diverse transgenic concepts like the constitutive expression of pathogenesis‐related proteins PR‐1a and PR‐5 (Zhu *et al*., 1996), the transfer of the resveratrol synthase gene (Thomzik *et al*., 1997), the expression of antimicrobial peptides (Osusky *et al*., 2005) or the inactivation of susceptibility genes by RNAi or CRISPR/Cas9 (Kieu *et al*., 2021; Sun *et al*., 2016) resulted in an increase in plant resistance against oomycetes under laboratory conditions.

Plants possess a highly efficient, two layered innate immune system that makes them resistant against most microbial pathogens (Jones and Dangl, 2006). The first layer of defence relies on the recognition of evolutionary conserved pathogen‐ or microbial‐associated molecular patterns (PAMPS or MAMPs) by pattern recognition receptors (PRRs). PAMPs or MAMPs are invariant structures broadly represented among microbial taxa and have essential roles in microbial physiology. Only an extremely select group of molecules have been found to function as PAMPs (Gaudet and Grey‐Owen, 2016). Recognition of pathogen‐associated molecular patterns (PAMPs) in the apoplast by PRRs initiates a complex signalling cascade leading to PRR‐triggered immunity (PTI). Adapted pathogens will partially suppress the first defence layer through the secretion of effector proteins that interfere with the signalling (Césari *et al*., 2014; Jones and Dangl, 2006). The second layer of plant defence, the effector triggered immunity (ETI), relies on the specific recognition of effectors by disease resistance genes (Jones and Dangl, 2006). This recognition leads to a strong defence response which is often associated with a local programmed cell death, the hypersensitive reaction. Since effectors are generally species‐ or isolate‐specific, this second layer of immunity is only efficient against isolates that carry the recognized effector, which is then called an avirulence gene (Césari *et al*., 2014).

The engineering of transgenic plants using the benefits of PTI and ETI without the limitations of the two layers of the innate system would be highly attractive and should allow the development of plants with broad and durable disease resistance. A visionary concept that corresponds to this idea was already proposed as ‘Avr/R strategy’ a few decades ago by de Wit (1992). The Avr/R strategy involves the transfer of an avirulence gene into a plant containing the corresponding resistance gene and its subsequent expression under the direction of a promoter that is rapidly and locally inducible by a wide range of fungal pathogens (dde Wit, 1992; Joosten and de Wit, 1999). This strategy was applied to the *Cf‐*9/Avr9 gene pair using a 273 base‐pair fragment of the pathogen‐inducible promoter Gst1 from potato (Martini *et al*., 1993). Although preliminary results showed enhanced resistance of tomato against two fungal pathogens, further optimization of the tight promoter regulation was suggested since promoter specificity was not fully restricted to the infection sites (Joosten and de Wit, 1999; Strittmatter *et al*., 1996).

An ideal pathogen‐inducible promoter would fulfil the following criteria for a biotechnological application as defined by Gurr and Rushton (2005). First, the promoter should be activated locally and in response to a broad set of pathogens and therefore allowing a broad pathogen resistance. Second, the promoter should be inactive under disease‐free conditions and should show no background activity. Third, the promoter should not be autoactivatable by the transgene and cause an undesired spread of promoter activation which results in a ‘runaway cell death’ phenomena.

Plant promoters consist of a core or minimal promoter region, a proximal promoter region and distal promoter regions. Distal promoter regions contain enhancer, silencer and insulator *cis‐* elements. The proximal promoter region contains *cis*‐elements bound by activator and repressor proteins. The core promoter generally spans from −50 bp to +50 bp relative to the transcription start site (TSS) and enables assembly of the transcription initiation complex. It contains a TATA box, TF IIB recognition elements, a Y patch and downstream promoter elements (DPEs). There is a spacer sequence of no less than 50 bp between the core promoter and the proximal promoter region (Yasmeen *et al*., 2023). Core promoters define the TSS, but their activity typically leads to only low levels of expression (Jores *et al*., 2021).

The modular organization of the proximal promoter regions with several different cis‐elements for tissue‐specificity, hormone sensitivity, abiotic‐ and biotic stress responsiveness and developmental processes is the reason for the complex expression pattern of many plant genes. The design of synthetic promoters composed of a core promoter region and an artificial proximal promoter region with a limited number of cis‐acting elements is an attractive approach to develop promoters with a desired specificity and low background activity. In pioneering work, the first synthetic pathogen‐inducible promoters were developed and the basic rules for synthetic promoter design were described (Rushton *et al*., 2002).

The key element of a synthetic promoter is the individual cis‐acting element which was either identified after a detailed analysis of an individual inducible promoter (Heise *et al*., 2002; Kirsch *et al*., 2001; Van de Löcht *et al*., 1990) or by a bioinformatic analysis of co‐regulated pathogen‐induced genes (Koschmann *et al*., 2012). The individual cis‐acting element will basically define the strength, background activity, spatial expression pattern and induction kinetic of a synthetic promoter. An increasing number of the individual cis‐acting elements will increase the promoter strength but simultaneously the background activity. Synthetic promoters composed of two different cis‐acting elements showed a promoter performance which is superior to the properties of a promoter which was constructed with the individual elements (Gurr and Rushton, 2005; Rushton, 2016; Rushton *et al*., 2002). The simple modular organization of a synthetic promoter in comparison with a natural promoter allows a rationale design and better adaptation to the specific needs of gene expression. Synthetic pathogen‐inducible promoters have been successfully used for reporter gene expression in *Arabidopsis thaliana* after fungal infection (Rushton *et al*., 2002), PAMP‐induction in a parsley protoplast system (Koschmann *et al*., 2012), bacteria resistance of tobacco (Niemeyer *et al*., 2014) and the development of nematode responsive soybean promoter (Liu *et al*., 2014).

The synthetic promoters 2xS‐2xD‐35S_minimal_ and 4xW2‐35S_minimal_ have been shown to be inducible after bacterial infection in tobacco (Niemeyer *et al*., 2014) and fungal infection in *A. thaliana* (Rushton *et al*., 2002). The 2xS‐2xD‐35S_minimal_ and 4xW2‐35S_minimal_ promoters are composed of dimers of the S‐box and the D‐box and a tetramer of the W2‐box, respectively, which were linked to the minimal promoter of the *35S* gene from the cauliflower mosaic virus (CaMV). The D‐box was isolated as a PAMP‐inducible cis‐element from the parsley *PR2* gene promoter (Rushton *et al*., 2002; van de Löcht *et al*., 1990) and the S‐box was described as a PAMP‐inducible cis‐element of the *ELI7.1* gene promoter (Kirsch *et al*., 2001; Rushton *et al*., 2002). The W2‐box from the parsley *PR1* gene (Rushton *et al*., 1996) proved its suitability for the design of a synthetic pathogen‐inducible promoter in *A. thaliana* (Rushton *et al*., 2002).

The NBS‐LRR resistance gene *R3a* of potato was cloned by a comparative genomics approach using synteny between the complex *R3a* locus of potato and the *I2* complex locus of tomato (Huang *et al*., 2005). To date, R3a function is only shown for two members of the *Solanaceae* family (*Solanum tuberosum*, *N. benthamiana*).

The *Phytophthora infestans Avr3a* gene, the corresponding avirulence gene of *R3a*, was identified with association genetics and was the first reported *P. infestans* avirulence effector. *Avr3a* encodes a protein that is recognized in the host cytoplasm, where it triggers *R3a*‐dependent cell death (Armstrong *et al*., 2005). Avr3a is an oomycete effector with an Arg‐any amino acid‐Leu‐Arg (RxLR) motif that is required for secretion (Wawra *et al*., 2017).

Avr3a has some features that make the effector very interesting and suitable for the Avr/R strategy. The avirulence gene *Avr3a* occurs in all *P. infestans* isolates worldwide in two forms, the *Avr3a* ^*K80I103*^ (*Avr3a* ^*KI*^) or as *Avr3a* ^*E80M103*^ (*Avr3a* ^*EM*^) allele. Avr3a is essential for the virulence of *P. infestans* (Bos *et al*., 2010), and a deletion or silencing variant of *Avr3a* has not been identified so far in field isolates. The *Avr3a* ^*KI*^ allele activates *R3a* resistance gene dependent innate immunity, whereas Avr3a^EM^ will not be recognized by R3a (Armstrong *et al*., 2005). Only the virulent allele *Avr3a* ^*EM*^ is present in the modern lineage, which results in the loss of resistance of *R3a*‐expressing potatoes. A structure–function analysis of both alleles revealed that amino acid K80 is important for R3a activation regardless of the polymorphism at residue 103. Substitutions of the AVR3a amino acid residue at Position 80 to all possible amino acids confirmed that this site is important for both the virulence and avirulence activities (Bos *et al*., 2009).

Here, we show that the *P. infestans* resistance of potato was improved by the R/Avr concept without a detrimental effect on the plants. In a two‐step process, the pathogen inducibility of the S‐D type promoter was optimized for potato by the identification of an ideal core promoter for the Solanacea crop and by increasing the number of cis‐elements to generate the 2xS‐4xD promoter. Mutated *Avr3a* alleles having reduced cell death‐inducing activity compared with the *av*irulent *Avr3a* ^*K*I^ allele were expressed with the optimized synthetic promoter. At the same time, the modified *Avr3a* alleles were still capable of activating innate immunity during pathogen infection.

## Results

### 35S minimal promoter is not suitable for the synthetic promoter design in potato

The co‐expression strategy of an *R*‐ and an *Avr* gene requires a highly pathogen specific‐inducible promoter which is locally expressed at the infection site and shows no or low background activity in non‐infected tissue. The usefulness and importance of the synthetic promoter 2xS‐2xD‐35S_minimal_ have been reported (Niemeyer *et al*., 2014). Therefore, the 2xS‐2xD‐35S_minimal_ and the 4xW2‐35S_minimal_ (Rushton *et al*., 2002) promoter were tested in potato for *P. infestans* responsiveness.

**Figure 1:**
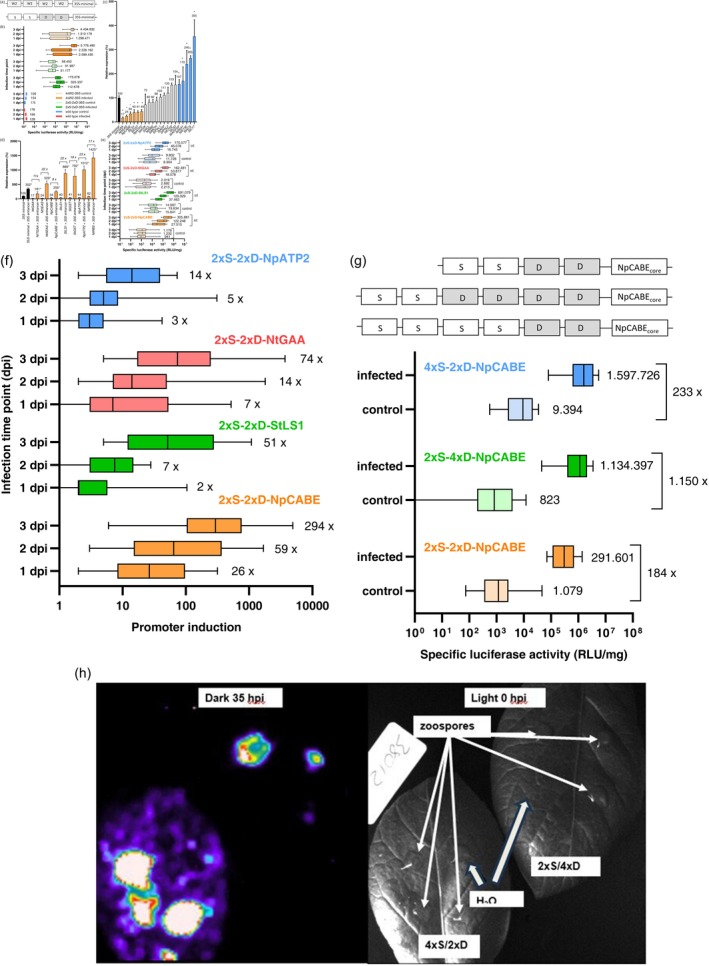
Development of the synthetic pathogen‐inducible 2xS‐4xD‐NpCABE_core_ promoter for potato. (a) Scheme of the synthetic promoters 4xW2‐35S_minimal_ and 2xS‐2xD‐35S_minimal_. The 4xW2‐35Sminimal promoter is composed of a tetramer of the cis‐acting element W2 (W2‐box) and the minimal promoter of the *35S* gene. The 2xS‐2xD‐35S_minimal_ promoter is composed of a dimer of the cis‐acting element S (S‐box), a dimer of the cis‐acting element D (box) and the minimal promoter of the 35S gene. The sequence information of the promoter elements is shown in Figure S4. (b) The synthetic 4xW2‐35S_minimal_ and 2xS‐2xD‐35S_minimal_ promoters show high background activity and low pathogen inducibility in transgenic potato lines. Median values of specific luciferase activity (RLU/mg) from 20 lines of each construct are shown. Leaves of transgenic reporter gene plants from the greenhouse were infected with *P. infestans* in a detached leaf assay and analysed 1, 2 and 3 dpi.  wild type = non‐transgenic cultivar. (c) Relative expression level of the luciferase gene in potato leaves driven by 20 core promoters from 20 Solanacea genes in a transient biolistic assay. Luciferase activity of the core promoter luciferase constructs was normalized against the activity of the 35S minimal promoter luciferase construct. Mean of four replicates of each construct is shown. The standard deviation of the mean is given by error bars. The asterisk (*) indicate a statistically significance of the data compared with the 35S minimal promoter according ANOVA analysis and a pair‐wise comparison with Tukey test (*t*‐test), *p* < 0.05. (d) Relative expression level of the luciferase gene in potato leaves driven by seven core promoters and the corresponding core promoter‐35S enhancer combinations in a transient biolistic assay. Luciferase activity of the core promoter and core promoter–enhancer constructs was normalized against the activity of the 35S minimal promoter luciferase construct. Mean of four replicates of each construct is shown. Induction factor by the enhancer is given as number above the core promoter–enhancer column. The standard deviation of the mean is given by error bars. The asterisk (*) indicates a statistically significance of the data compared with the corresponding core promoter according ANOVA analysis and a pair‐wise comparison with Tukey test (*t*‐test), *p* < 0.05. (e) Median activity of 2xS‐2xD‐core promoters in potato lines after *P. infestans* infection. Transgenic potato reporter gene lines transformed with the 2xS‐2xD‐NtTGAA_core_, 2xS‐2xD‐NpCABE_core_, 2xS‐2xD‐StLS1_core_ and 2xS‐2xD‐NpATP2_core_ promoter in combination with the luciferase gene were tested in an *in vitro* assay for late blight responsiveness and analysed 1, 2 and 3 dpi. Plants were treated with water as a control. For each construct 20 lines with 3 replicates were analysed and the median of luciferase expression calculated. Specific luciferase activity (RLU/mg) is shown. (f) Median induction of 2xS‐2xD‐core promoters in potato lines after P. infestans infection. Transgenic potato reporter gene lines transformed with the 2xS‐2xD‐NtTGAA_core_, 2xS‐2xD‐NpCABE_core_, 2xS‐2xD‐StLS1_core_ and 2xS‐2xD‐NpATP2_core_ promoter in combination with the luciferase gene were tested in an *in vitro* assay for late blight responsiveness at 1, 2 and 3 dpi. For each construct, 19–25 lines with three replicates were analysed. The median of data is shown. (g) Differential activity of the synthetic promoters 2xS‐2xD‐NpCABE_core_, 2xS‐4xD‐NpCABE_core_ and 4xS‐2xD‐NpCABE_core_ in transgenic potatoes. Top: Schematic representation of the synthetic promoters 2xS‐2xD‐NpCABE_core_, 2xS‐4xD‐NpCABE_core_ and 4xS‐2xD‐NpCABE_core_ is shown. Bottom: Transgenic potato reporter gene lines transformed with the 2xS‐2xD‐NpCABE_core_, 2xS‐4xD‐NpCABE_core_ and 4xS‐2xD‐NpCABE_core_ promoter in combination with the luciferase gene were tested in an *in vitro* assay for late blight responsiveness and analysed 3 dpi. Plants were treated with water as a control. For each construct 19–25 lines with three replicates were analysed. The median of luciferase expression and the median of induction was calculated. Specific luciferase activity (RLU/mg) is shown. (h) Local expression of the luciferase gene in transgenic potato leaves after *P. infestans* infection mediated by the 4xS‐2xD‐NpCABE_core_ and 2xS‐4xD‐NpCABE_core_ promoter detected by a CCD camera. The 2xS‐4xD‐NpCABE_core_ promoter showed highest specificity at the infection site, whereas the 4xS‐2xD‐NpCABE_core_ promoter revealed background activity in the leaf 35 h after inoculation. Light photograph of the leaves showed droplets of zoospores and water at the inoculation sites marked with an arrow immediately after inoculation.

The synthetic promoters 2xS‐2xD‐35S_minimal_ and 4xW2‐35S_minimal_ were combined with the luciferase reporter gene of *Photinus pyralis* and transformed into potato (Figure 1a). The analysis of the reporter gene plants revealed that the background activity of the synthetic promoters was unusually high in non‐infected leaves (Figure 1b) compared with data from transgenic sugar beet lines transformed with the same 2xS‐2xD‐35S_minimal_ construct (Figure S1) and 4xW2‐35S_minimal_ reporter gene plants of *A. thaliana* (Rushton *et al*., 2002). After *P. infestans* infection of potato leaves in a detached leaf assay the 2xS‐2xD‐35S_minimal_ and the 4xW2‐35S_minimal_ reporter gene plants showed only a very weak induction of the reporter gene (Figure 1b). With a threefold, fourfold and threefold median pathogen inducibility at 1, 2 and 3 dpi the 2xS‐2xD‐35S_minimal_ promoter showed only a weak inducibility. The inducibility of the 4xW2‐35S_minimal_ promoter was even less and could only be demonstrated on day 1. In contrast, the 2xS‐2xD‐35S_minimal_ promoter showed a low background activity in non‐infected sugar beet plants and a strong induction after infection with the fungal pathogen *Cercospora beticola*. With a median induction factor of 2, 6, 35, 31 and 80, the 2xS‐2xD‐35S_minimal_ promoter was highly inducible on days 1, 2, 3, 4 and 6 in sugar beet leaves (Figure S1). The 35S minimal promoter spanning the region −46 to +8 of the *35S* gene was less suitable for synthetic promoter design in potato as in other plants.

### Core promoter sequences of *Solanaceae* genes show different basic promoter activities

Bhullar *et al*. (2003) described a significant reduction in the promoter activity of the 35S promoter when the minimal promoter (−46 to +1) was replaced by heterologous plant minimal promoters. To identify a more suitable minimal or core promoter, 20 core promoter sequences from different *Solanacea* species were selected. Core promoter sequences of genes from *Nicotiana plumbaginifolia*, *Nicotiana tabacum* and *Solanum tuberosum*, which were in the range from approximately position −50 to +15 to the transcriptional start point of the genes were isolated and analysed. To obtain a wide variety of sequences, genes with different functions and expression behaviour were selected. The genes encoded proteins from metabolism and genes induced either by light, biotic or abiotic stress. From *Nicotiana plumbaginifolia* the core promoter sequences of the chlorophyll a/b‐binding protein (*NpCABE*) and the beta subunit of the mitochondrial ATP synthase (*NpATP2*) genes were used. From *Nicotiana tabacum*, the core promoter sequences of a TGA transcription factor (*NtTGAA*), a 5‐epi‐aristolochene synthase (*Nt5EAS*), the small subunit of the ribulose bisphosphate carboxylase (*NtRBS*), an endochitinase (*NtCHI2*), a nitrate reductase (*NtNIA1*) and 1,3‐beta glucanase (*NtE13E*) genes were selected. From *Solanum tuberosum* the core promoter regions of the light‐induced LS1 gene (*StLS1*), a glutathione S‐transferase (*StGST*), two proteinase inhibitors (*StIP2K*, *StCID*), the p‐coumaroyl‐CoA‐ligase (*St4CL*), two phenylalanine ammonia‐lyases (*StPAL1*, *StPAL2*), a metallo carboxypeptidase inhibitor (*StMCPI*), the small and the large subunit of the ADP‐glucose pyrophosphorylase (*StADP* and *StADPGP*), the 1‐aminocyclopropane‐1‐carboxylate synthase (*StACS*) and cold‐stress induced *StC17* genes were selected.

The 20 core promoters were combined with the luciferase gene. After transient expression of the reporter gene constructs in potato leaves by biolistic transformation, the luciferase expression driven by the core promoters was measured. The six core promoters NtTGAA, Nt5EAS, NpCABE, StLS1, StGST and NpATP2 showed a statistically reduced activity compared with the 35S minimal promoter. In contrast the five core promoters of the genes *StADPGP*, *NtNIA1*, *NtE13E*, *StACS* and *StC17* caused a statistically increased reporter gene activity (Figure 1c).

To rule out that the reduced core promoter activity is caused by a loss of core promoter function, the six core promoters NtTGAA, Nt5EAS, NpCABE, StLS1, StGST and NpATP2 were combined with the 35S promoter enhancer. As a control the 35S enhancer was added to the 35S minimal promoter to reconstruct the 35S promoter. In addition, the NtRBS core promoter, which showed a similar activity to the 35S minimal promoter, was combined with the 35S enhancer. The core promoters and the corresponding combinations of a core promoter and the 35S enhancer as a distal element were transiently tested for the expression of the luciferase gene. All six of the core promoter–enhancer constructs showed 8‐ to 23‐fold stronger luciferase activity compared with the single core promoter constructs (Figure 1d). This experiment revealed that the core promoters of the *NtTGAA*, *Nt5EAS*, *NpCABE, StLS1*, *StGST* and *NpATP2* genes are functioning, and that the lower basic promoter activity compared with the 35S minimal promoter is sequence dependent and not caused by a loss of core promoter function. Furthermore, the individual core promoters differentially influenced the strength of the core promoter‐35S enhancer constructs revealing that the core promoter is important for the quantitative promoter strength.

### The core promoter contributes to strength and pathogen inducibility of a synthetic promoter

Having shown that the core promoter sequence influences the promoter strength in a transient assay, the influence of the core promoter on the property of a pathogen‐inducible synthetic promoter was analysed in transgenic plants. The core promoter sequences of the *NtTGAA*, *NpCABE*, *StLS1* and *NpATP2* genes were selected from the group of six core promoters with reduced promotor activity for further analysis. The chosen core promoter sequences showed different levels of core promoter activity (Figure 1c) and differed in promoter strength after combination with the 35S enhancer sequence (Figure 1d). This selection made it possible to assemble a diverse group of core promoters for the next step of analysis.

The four core promoter sequences were combined with the dimer of the S‐box and a dimer of the D‐box, resulting in the synthetic promoters 2xS‐2xD‐NtTGAA_core_, 2xS‐2xD‐NpCABE_core_, 2xS‐2xD‐StLS1_core_ and 2xS‐2xD‐NpATP2_core_. To analyse the effect of the core promoters, the 2xS‐2xD promoters were combined with the luciferase gene and stably transformed into potato. Twenty independent lines were generated with each construct and the ability of the promoters to control gene expression were determined 1, 2 and 3 dpi after infection of potato leaves with *P. infestans*. The 2xS‐2xD promoters differed in their background activity, promoter strength and pathogen inducibility. The median promoter strength of the 2xS‐2xD‐NpCABE_core_ and 2xS‐2xD‐StLS1_core_ promoters was 2,5‐ and threefold higher after *P. infestans* infection compared with the 2xS‐2xD‐NtTGAA_core_ and 2xS‐2xD‐NpATP2_core_ promoters at 2 dpi. After 3 dpi the median promoter activity of the of the 2xS‐2xD‐NpCABE_core_ and 2xS‐2xD‐StLS1_core_ promoters were two‐ and fourfold higher compared with the 2xS‐2xD‐NtTGAA_core_ and 2xS‐2xD‐NpATP2_core_ promoters. The highest median activity of all promoters was measured for the 2xS‐2xD‐StLS1_core_ promoter at 3 dpi (Figure 1e).

The 2xS‐2xD‐NpCABE_core_ promoter showed the lowest median background activity of all four promoters in the water control at 1, 2 and 3 dpi. The low median background activity and the high median pathogen‐induced activity gave the 2xS‐2xD‐NpCABE_core_ promoter the highest pathogen inducibility and pathogen specificity of all tested promoters. With a 26‐, 59‐ and 294‐fold median pathogen inducibility at 1, 2 and 3 dpi this property gave the 2xS‐2xD‐NpCABE_core_ promoter an outstanding characteristic (Figure 1f). Based on these results the NpCABE core promoter was selected for further synthetic promoter design. In summary, the promoter analysis revealed that the core promoter strongly influenced the quantitative strength, specificity and pathogen inducibility of a synthetic promoter in a transgenic plant.

### Development of the optimized 2xS‐4xD‐NpCABE _core_ promoter

The expression strength and inducibility of a synthetic promoter will be determined by the copy number of the *cis*‐elements as shown by Rushton *et al*. (2002). To optimize the 2xS‐2xD‐NpCABE_core_ promoter, the dimers of the S‐box and D‐box were replaced by tetramers of the S‐box and D‐box, respectively. The resulting 4xS‐2xD‐NpCABE_core_ and 2xS‐4xD‐NpCABE_core_ promoters were combined with the luciferase gene, transformed into potato and tested with the 2xS‐2xD‐NpCABE_core_ promoter plants for *P. infestans* inducibility 3 dpi after infection.

The median promoter strength of the 2xS‐4xD‐NpCABE_core_ and 4xS‐2xD‐NpCABE_core_ lines were 4‐ and 5,5‐fold higher after *P. infestans* infection compared with the 2xS‐2xD‐NpCABE_core_ promoter lines. The median background activity of the 4xS‐2xD‐NpCABE_core_ lines was ninefold higher compared with the 2xS‐4xD‐NpCABE_core_ promoter lines, whereas the mean background activity of the 2xS‐4xD‐NpCABE_core_ lines was slightly less than the background of the 2xS‐2xD‐NpCABE_core_ promoter lines. The median pathogen inducibility of the 2xS‐4xD‐NpCABE_core_ lines was five‐ and sixfold higher compared with the 4xS‐2xD‐NpCABE_core_ and 2xS‐2xD‐NpCABE_core_ lines (Figure 1g).

To analyse and compare the spatial activity of the 4xS‐2xD‐NpCABE_core_ and 2xS‐2xD‐NpCABE_core_ promoters, selected lines transformed with the 4xS‐2xD‐NpCABE_core_ and 2xS‐4xD‐NpCABE_core_ promoter constructs were transferred to the greenhouse. Potato leaves were infected with droplets of *P. infestans* zoospores in a detached leaf assay and luciferase expression was detected with a CCD camera 35 h after inoculation. As shown in Figure 1h the 2xS‐4xD‐NpCABE_core_ promoter was specific and locally activated by *P. infestans* but not by a water droplet which was added as a control to the leaf. Luciferase activity was not detectable in the leaf tissue outside the *P. infestans* inoculation zone in accordance with the low background activity measured for this promoter. The 4xS‐2xD‐NpCABE_core_ promoter was locally and highly activated by *P. infestans* but minor luciferase activity was detectable too in the leaf tissue outside the inoculation zones explaining the higher background activity of this promoter.

In summary, the tetramer of the D‐box of the 2xS‐4xD‐NpCABE_core_ promoter increased the promoter strength and pathogen inducibility compared with the 2xS‐2xD‐NpCABE_core_ promoter but did not increase the background activity. The tetramer of the S‐box of the 4xS‐2xD‐NpCABE_core_ promoter increased the promoter strength but also the background activity of the promoter compared with the 2xS‐2xD‐NpCABE_core_ promoter. These findings suggest that the 2xS‐4xD‐NpCABE_core_ promoter is the best suited synthetic promoter to mediate *P. infestans* inducibility in potato.

### Pathotype analysis of *P. Infestans* strain Gross‐Luesewitz

The pathotype or race specificity of the *P. infestans* isolate Gross‐Luesewitz, which had been isolated in Germany, was analysed with a differential set of *Rpi*‐gene expressing potato lines (Mastenbroek, 1952) in a field trial. Potato lines with the *Rpi*‐genes *R1, R2, R3, R4, R5, R6, R7, R8, R9, R10* and *R11* were grown in plots, infected with the *P. infestans* isolate and scored at 3 time points for resistance. All tested *Rpi*‐genes except for R8 and R9 were defeated by the isolate showing the race specificity 1, 2, 3, 4, 5, 6, 7, 10, 11 (Figure S2).

### Molecular stack of *R3a* resistance gene and *Avr3a* ^*KI*^ avirulence gene in potato

**Figure 2:**
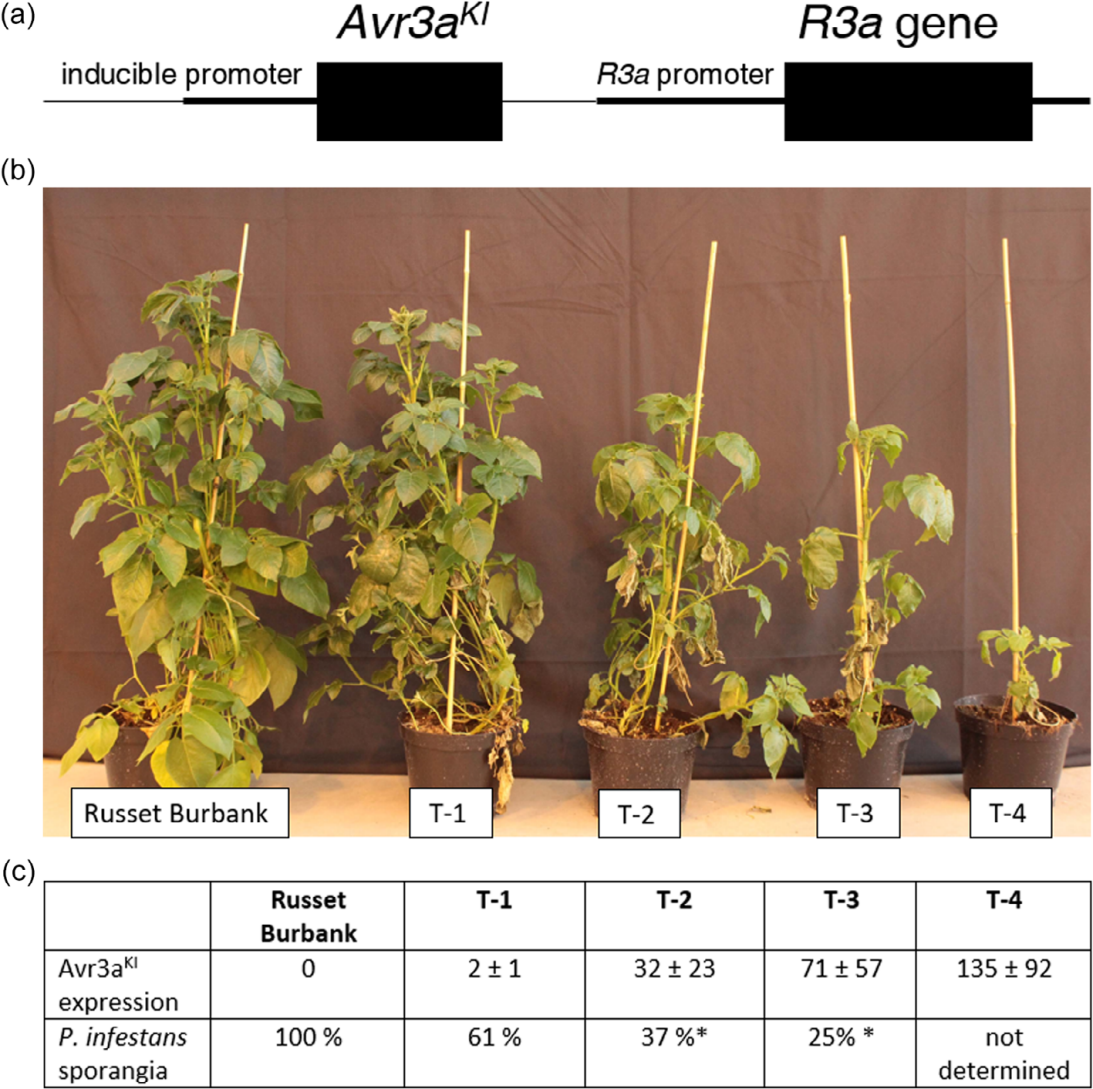
*R3a‐Avr3a* co‐expression in adult plants of the cultivar Russet Burbank resulted in enhanced late blight resistance with a detrimental effect to the plant phenotype. (a) Schematic representation of the transformed cassette of the *Avr3a* ^*KI*^ gene under control of the synthetic promoter 2xS‐4xD‐NpCABE_core_ and the *R3a* gene. (b) Phenotype of the transgenic lines T‐1, T‐2, T‐3 and T‐4 in the greenhouse compared with Russet Burbank. (c) Comparison of Avr3a gene expression in the transformed lines with the reduction in sporangia production after *P. infestans* infection. Expression of the *Avr3a* ^*KI*^ gene in potato leaves was measured by qRT‐PCR analysis. *Avr3a* ^*KI*^ gene expression was normalized against expression of the potato house‐keeping gene *StMCB75*. *n* = 4, ± indicates standard deviation. Late blight resistance was determined as sporangia number in a detached leaf assay (DLA).

A molecular stack of the native *R3a* resistance gene of potato and the *Avr3a* ^*KI*^ gene was constructed in a binary vector and transformed into the potato cultivar Russet Burbank which lacks an *R3a* gene. The expression of the *R3a* gene was under control of the endogenous *R3a* promoter and the *Avr3a* expression was controlled by the pathogen‐inducible, synthetic promoter 2xS‐4xD‐NpCABE_core_ (Figure 2a).

Transgenic *R3a‐Avr3a* ^*KI*^ lines were generated and transferred into the greenhouse as rooted *in vitro* plants. First, the plantlets developed well in soil, showing no difference to the non‐transgenic Russet Burbank. However, 3–4 weeks after transfer to the greenhouse, spontaneous necrosis of leaf tissue, abortion of leaves and stunted growth of the plants were observed. The *R3a‐Avr3a* ^*KI*^ lines T‐1, T‐2, T‐3 and T‐4, which were analysed in detail, showed a reproducible gradient of disintegration (Figure 2b). Line T‐1 showed 30% size reduction compared with the non‐transformed Russet Burbank and spontaneous leaf necrosis. The lines T‐2 and T‐3 showed 50% size reduction and single leaf fall. The line T‐4 showed 80% size reduction and severe leaf fall.

A qRT‐PCR analysis revealed that *Avr3a* ^*KI*^ transcripts were detectable at different level in the leaves of the uninfected *R3a‐Avr3a* ^*KI*^ lines (Figure 2c). The level of the negative phenotype of adult *R3a‐Avr3a* ^*KI*^ plants correlated with the expression of the *Avr3a* ^*KI*^ gene. Whereas *Avr3a* ^*KI*^ expression was not detectable in the leaves of Russet Burbank the *Avr3a* ^*KI*^ transcript level increased in the transgenic lines in the order T‐1, T‐2, T‐3 and T‐4. Expression of the *Avr3a* ^*KI*^ gene was also detectable in healthy young greenhouse plants (Figure S3) and the *Avr3a* expression was even higher than in adult plants, but the development of young plants was not harmed. This indicated that the synthetic promoter activity is development stages dependent.

The late blight resistance of the *R3a‐Avr3a* ^*KI*^ lines was determined with a detached leaf assay. In comparison with Russet Burbank, the adult transgenic lines showed enhanced resistance as shown by counting the sporangia number of *P. infestans* which were formed on the infected leaves. The lines T‐1, T‐2 and T‐3 showed only 61%, 39% and 25% of the sporangia number produced by Russet Burbank leaves (Figure 2c). The level of resistance improvement correlates with the level of *Avr3a* ^*KI*^ gene expression in uninfected leaves. The severe spontaneous necrosis of T‐4 leaves prevented the determination of resistance of this line.

The negative response upon co‐expression of 2xS‐4xD‐NpCABE_core_::*Avr3a* ^*KI*^ with an R3a‐gene in Russet Burbank raised the idea to test the concept in potato plants with an endogenous R3a gene. The 2xS‐4xD‐NpCABE_core_::*Avr3a* ^*KI*^ cassette was transformed into the potato cultivar Baltica which contains an endogenous *R3a* gene. The Baltica lines which had been transformed with 2xS‐4xD‐NpCABE_core_::*Avr3a* ^*KI*^ showed also spontaneous leaf necrosis and gradations of stunted growth (Figure S4) as the transformed Russet Burbank. The transgenic Baltica plants with a conspicuous phenotype revealed enhanced resistance to late blight (Table S2). Lines without a phenotype displayed no enhanced resistance.

Although the results proved that late blight resistance of potato cultivars Russet Burbank and Baltica could be improved by induced expression of the *Avr3a* ^*KI*^ gene the detrimental effect of the approach was still obvious and prevented an agricultural application.

### Modification of Avr3a activity

The pathogen‐induced expression of the *Avr3a* ^*KI*^ gene in combination with a co‐expressed *R3a* gene showed promising results but revealed as well that the basic expression of the *Avr3a* ^*KI*^ gene was still too high in non‐infected adult plants. To avoid an unplanned activation of the defence system, the *Avr3a* ^*KI*^ allele was replaced by an *Avr3a* allele with less avirulence activity.

**Figure 3:**
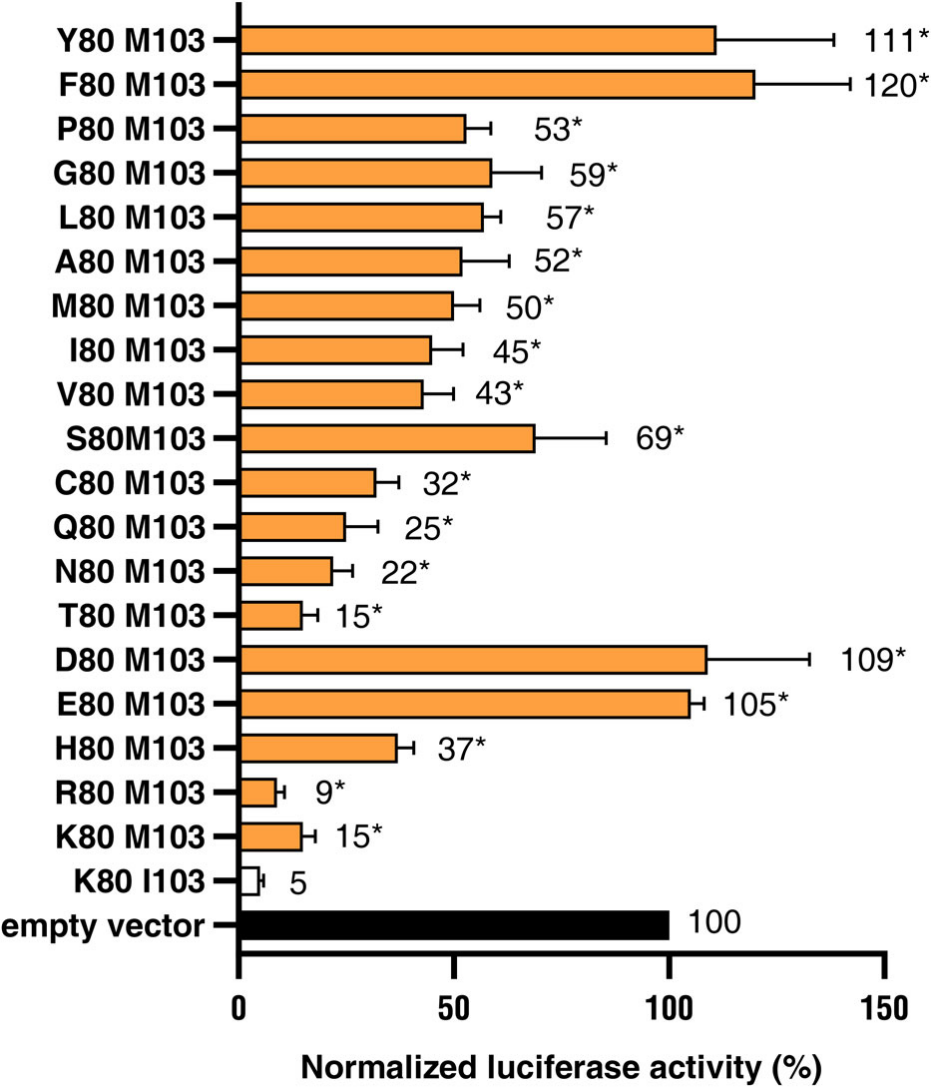
Allelic variants of the *Avr3a* gene trigger different levels of cell death in potato leaves of the cultivar Hermes. *Avr3a* variants under the control of the doubled 35S promoter trigger different levels of cell death after transient expression in potato leaves of the R3a cultivar Hermes. The mean of three independent experiments with six replicates each is shown for each allele. Leaf discs from leaves of 6 to 8 weeks old greenhouse plants of the cultivar Hermes were cut with a cork borer and mingled. For each biological replicate, eight leaf discs were placed on an agar plate and bombarded. The normalized luciferase activity of the empty vector was set to 100. The tested *Avr3a* alleles differ in the amino acids at Position 80 and 103. The standard error of mean from three experiments is given by error bars. Data of all alleles (grey columns) are significantly different to *Avr3a* ^*KI*^ (white column) according to an ANOVA and Tukey test (pair‐wise *t*‐test, *p* < 0.05).

In this study a gain‐of‐function analysis of Avr3a was done in potato using a transient biolistic transformation system. The *Avr3a* allele of interest was co‐transferred with the *Photinus pyralis* luciferase gene into potato leaves of the cultivar Hemes which expressed the corresponding *R3a* gene. The induction of Avr3a‐mediated cell death reduced the expression of the *P. pyralis* luciferase gene and allowed the reporter gene activity to be used as a readout for the Avr3a activity. The transformation efficiency was normalized against the expression of the *Renilla reniformis* luciferase gene which was co‐transformed independently but simultaneously with the *Avr3a* allele into the potato leaves. The transfer of the native avirulent allele *Avr3a* ^*KI*^ resulted in only 5% of normalized luciferase activity compared with the virulent allele *Avr3a* ^*EM*^ and the vector control without an *Avr3a* allele (Figure 3). This result showed that the biolistic transformation technique allowed a quantitative detection of Avr3a‐R3a triggered cell death in combination with the dual luciferase system.

Starting from the virulent *Avr3a* ^*EM*^ sequence, the amino acid E at position 80 was substituted by 18 of the 20 natural amino acids. The *Avr3a* alleles with the amino acid exchanges at position 80 were tested for R3a dependent cell death induction. The *Avr3a* ^*DM*^ allele which has like *Avr3a* ^*EM*^ a negatively charged amino acid at position 80 triggered no cell death like Avr3a^EM^. Furthermore, the two aromatic amino acids F and Y resulted in a loss of cell death induction of the alleles *Avr3a* ^*FM*^ and *Avr3a* ^*YM*^.

The remaining 15 *Avr3a* alleles ‐ *Avr3a* ^*HM*^, *Avr3a* ^*KM*^, *Avr3a* ^*RM*^, *Avr3a* ^*CM*^, *Avr3a* ^*NM*^, *Avr3a* ^*PM*^, *Avr3a* ^*QM*^, *Avr3a^TM^, Avr3a* ^*AM*^, *Avr3a* ^*GM*^, *Avr3a* ^*IM*^, *Avr3a* ^*LM*^, *Avr3a* ^*MM*^ *and Avr3a* ^*VM*^ triggered less cell death compared with *Avr3a* ^*KI*^. Except for *Avr3a* ^*RM*^, the differences between the *Avr3a* alleles and *Avr3a* ^*K*I^ were statistically significant in the mean of 3 trials. The level of normalized luciferase activity ranged from 9% to 69% compared with 5% of *Avr3a* ^*KI*^.

The alleles *Avr3a* ^*RM*^ and *Avr3a* ^*KM*^ with the basic amino acids R and K at Position 80 showed a normalized luciferase activity of 9 and 15% and are the strongest cell death‐inducing alleles beside *Avr3a* ^*KI*^. The allele *Avr3a* ^*HM*^ with the basic amino acid H triggered still a clear cell death but with a significantly higher luciferase activity of 37%. The polar amino acids T, N, Q, C, M and S conferred a broad range of cell death induction to the *Avr3a* allele as shown by a normalized luciferase activity of 15, 22, 25, 32, 50 and 69%, respectively. The cell death induction of *Avr3a* alleles with the aliphatic amino acids V, I, A, L and G was nearly in the middle between the avirulent *Avr3a* ^*KI*^ and the virulent *Avr3a* ^*EM*^ allele with a normalized luciferase activity of 43, 45, 52, 57 and 59%, respectively. The amino acid P was also in this order of magnitude in the allele *Avr3a* ^*PM*^ with a luciferase activity of 53%.

### Enhanced late blight resistance by expression of modified *Avr3a* alleles

Three *Avr3a* alleles with a moderate level of cell death‐inducing activity were selected for pathogen‐induced expression in transgenic potato plants. Compared with the avirulent allele *Avr3a* ^*K*I^ (95% cell death), the cell death‐inducing activity of *Avr3a* ^*IM*^, *Avr3a* ^*LM*^ and *Avr3a* ^*GM*^ was 55%, 43% and 41%, respectively. The three *Avr3a* alleles were combined with the synthetic 2xS‐4xD‐NpCABE_core_ promoter and stable transformed into the potato cultivar Hermes.

**Figure 4:**
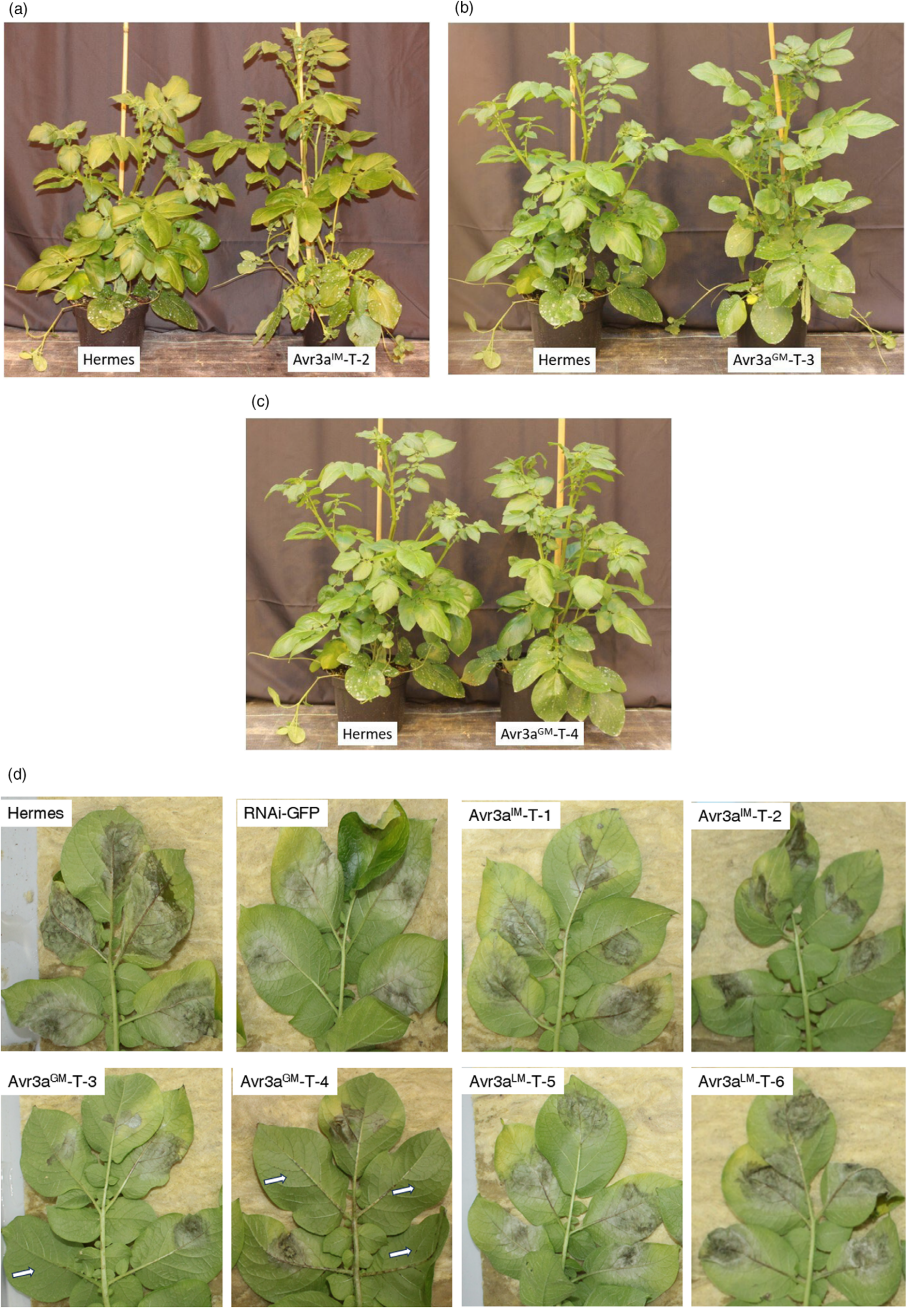
Transgenic potato lines Avr3a^IM^‐T‐2, Avr3a^GM^‐T‐3 and Avr3a^GM^‐T‐4 are phenotypically inconspicuous compared with the cultivar Hermes and show enhanced resistance. (a) Adult plant of the transgenic line Avr3a^IM^‐T‐2 showed the same phenotype as the non‐transformed cultivar Hermes in the greenhouse. (b) Adult plant of the transgenic line Avr3a^GM^‐T‐3 showed the same phenotype as the non‐transformed cultivar Hermes in the greenhouse. (c) Adult plant of the transgenic line Avr3a^GM^‐T‐4 showed the same phenotype as the non‐transformed cultivar Hermes in the greenhouse. (d) Enhanced late blight resistance of leaves from the lines Avr3a^IM^‐T‐2, Avr3a^GM^‐T‐3, Avr3a^GM^‐T‐4 in comparison with leaves of the cultivar Hermes, a transgenic control (RNAi‐GFP), and the lines Avr3a^IM^‐T‐1, Avr3a^LM^‐T‐5, Avr3a^LM^‐T‐6. The top five leaflets of each potato leaf were locally inoculated with drops of zoospores (20 μL with 600 zoospores). The first leaflet was inoculated with two drops and the second to fifth leaflets with one drop. The number and size of the lesions on the inoculated leaflets are reduced for line Avr3a^GM^‐T‐3 and Avr3a^GM^‐T‐4. Photographs were taken at 5 dpi. Inoculation sites which did not result in successfully infection are marked with white arrows.

Eight independent transgenic potato lines of each modified *Avr3a* gene were transferred twice to the greenhouse. The lines were analysed for phenotypic abnormality and oomycete resistance. Except for two lines, all lines showed the same vegetative growth phenotype as Hermes. One *Avr3a* ^*IM*^ line showed some growth retardation and the leaves of one *Avr3a* ^*LM*^ line revealed some micro‐necrotic lesions. However, these plants were viable too. A negative phenotype was not detectable for lines were *Avr3a* ^*GM*^ was transferred as shown for Avr3a^GM^‐T‐3 (Figure 4b) and Avr3a^GM^‐T‐4 (Figure 4c).  Also, the phenotype of the majority of Avr3a^IM^ lines was inconspicuous as shown for Avr3a^IM^‐T‐2 (Figure 4a). 

Both sets of plants were tested several times for late blight resistance in a detached leaf assay. The race specificity of the isolate had been determined before in field trials with a set of differential *Rpi*‐lines (Figure S2). Resistance was determined by counting the sporangia produced on the infected leaves 6 days post inoculation (dpi). The sporangia production is especially important with respect to the epidemiology of *P. infestans*. The highest level of resistance improvement was observed for the line Avr3a^GM^‐T‐4. This line showed a visible reduction in disease symptoms (Figure 4d) and on average only 40% sporangia production compared with the non‐transgenic cultivar Hermes and a transgenic control in five independent resistance assays (Table 1). Successful infection was frequently inhibited for some inoculated leaflets of line Avr3a^GM^‐T‐3 and Avr3a^GM^‐T‐4 in comparison with the controls as show in Figure 4d. The line Avr3a^IM^‐T‐2 and the line Avr3a^GM^‐T‐3 as well showed enhanced late blight resistance, as determined by having only 68% and 56% sporangia production (Figure 4d, Table 1).

**Table 1:** Infected potato leaves of Avr3a^IM^‐T‐2, Avr3a^GM^‐T‐3 and Avr3a^GM^‐T‐4 showed reduced production of *P. infestans* sporangia in comparison with Hermes and a transgenic control (RNAi‐GFP)

	Plant set 1 Sporangia (%) assay			Plant set 2 Sporangia (%) assay		Sporangia (%) Mean of 5 assays ± StDev
	1	2	3	4	5	
Hermes control	100	100	100	100	100	100 ± 0
RNAi‐GFP transgenic control	n.d.	n.d.	n.d.	98	108	103^n.s.^ ± 7 *p* < 0.3
Avr3a^IM^‐T‐1	22	89	71	105	86	74.6^n.s.^ ± 31.8 *P* < 0.11
Avr3a^IM^‐T‐2	30	59	96	91	62	67.6* ± 26.8 *p* < 0.03
Avr3a^GM^‐T‐3	26	65	77	87	26	56.2** ± 28.7 *p* < 0.009
Avr3a^GM^‐T‐4	25	31	63	54	28	40.2*** ± 17.1 *p* < 0.00005
Avr3a^LM^‐T‐5	44	39	81	88	130	76.4^n.s.^ ± 37 *p* < 0.19
Avr3a^LM^‐T‐6	96	153	n.d.	159	82	122.5^n.s.^ ± 39.2 *p* < 0.23

The sporangia were washed off from the infected potato leaves (*n* = 8) at 6 dpi and the number of sporangia were determined. The number of sporangia from Hermes was set to 100 for each experiment. In total, five independent resistance assays were performed with two different sets of plants. The line Avr3a^GM^‐T‐4 showed the highest reduction in sporangia formation in all five assays. Differences between samples were assessed by using analysis of variance (ANOVA) and a pair‐wise *t*‐test. Asterisk = Significant difference to Hermes according to pair‐wise *t*‐test, *p < 0.05, **p < 0.01, ***p < 0.001, n.d. = not determined, n.s. = not significant.

No significant resistance improvement was detectable for line Avr3a^IM^‐T‐1 and the lines *Avr3a* ^*LM*^‐T5 and *Avr3a* ^*LM*^‐T6 (Figure 4d, Table 1). In summary, these results indicate that a reduction in cell death‐inducing activity of the *Avr3a* gene is a promising strategy to circumvent the detrimental effects of uncontrolled *R‐Avr* gene expression and to enhance late blight resistance.

### *Avr3a* gene expression correlates with enhanced resistance

The expression of the recombinant *Avr3a* gene of the six transgenic lines Avr3a^IM^‐T‐1, Avr3a^IM^‐T‐2, Avr3a^GM^‐T‐3, Avr3a^GM^‐T‐4, Avr3a^LM^‐T‐5 and Avr3a^LM^‐T‐6 and Hermes was measured by qRT‐PCR analysis and normalized against the expression of the potato gene *StMCB75*. Leaves from greenhouse plants were infected with *P. infestans* in a DLA and RNA was extracted from inoculated and control leaves 0, 1, 2, 3 and 4 dpi.

**Figure 5:**
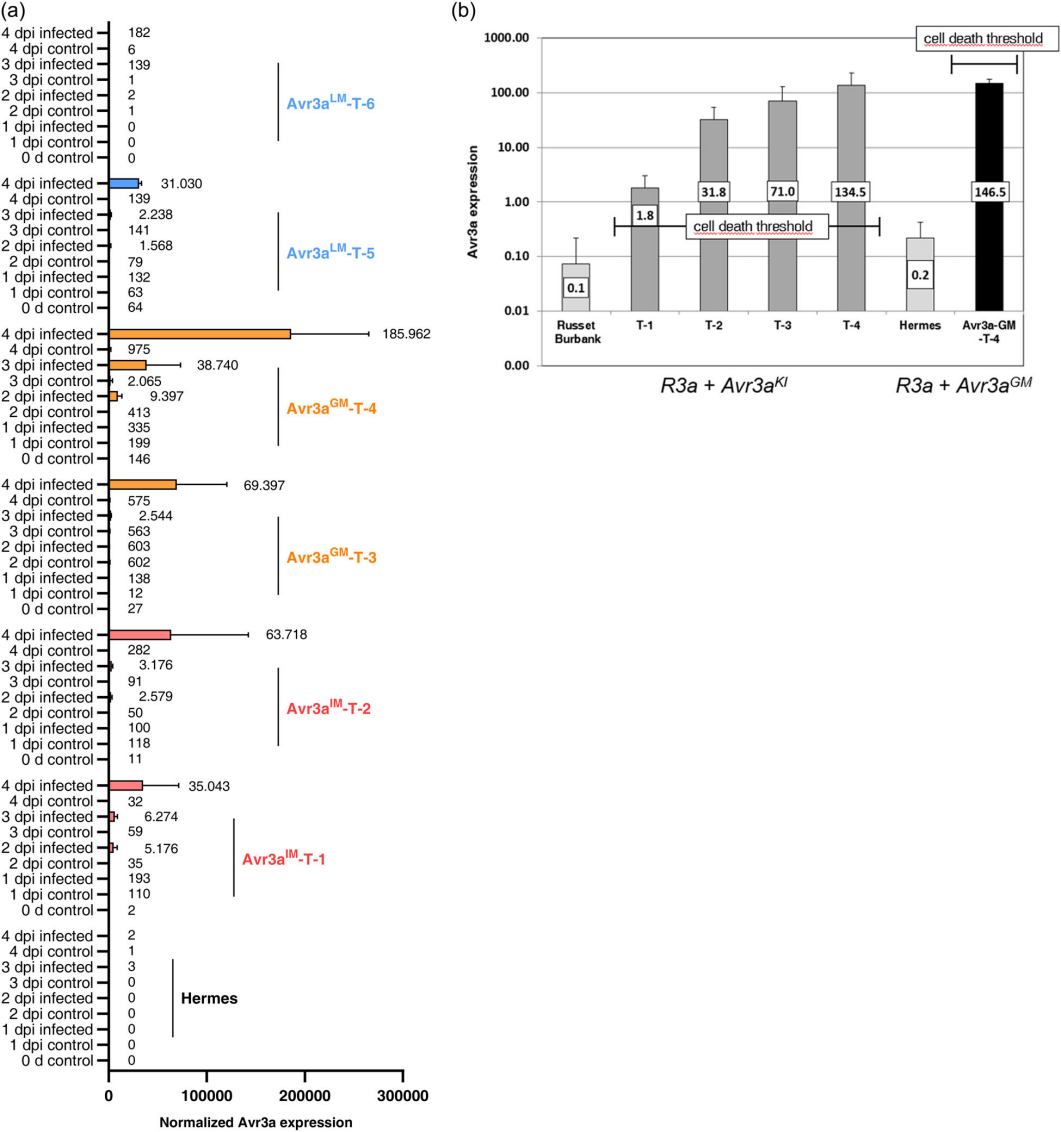
Expression analysis of the *Avr3a* gene in *P. infestans* infected and control leaves of transgenic potatoes by qRT‐PCR. (a) The expression of the *Avr3a* ^*IM*^, *Avr3a* ^*GM*^ and *Avr3a* ^*LM*^ alleles of the transgenic lines Avr3a^IM^‐T‐1, Avr3a^IM^‐T‐2, Avr3a^GM^‐T‐3, Avr3a^GM^‐T‐4, Avr3a^LM^‐T‐5 and Avr3a^LM^‐T‐6 were determined from uninfected and infected leaves by qRT‐PCR. The transgenic line with the best late blight resistance, Avr3a^GM^‐T‐4, showed the highest expression of the recombinant *Avr3a* gene after infection at 2, 3 and 4 dpi. RNA was isolated from four biological replicates of each treatment and time point for each line. *Avr3a* expression was normalized against the potato gene *StMCB75* and expression level given by numbers. *n* = 4. dpi = days postinoculation, ± indicates standard deviation. (b) Different threshold for cell death induction in the Avr3a^KI^ lines and the Avr3a^GM^‐T‐4 line was detected by expression analysis of non‐infected plants. The expression data of the *Avr3a* ^*KI*^ allele in non‐infected leaves from transgenic line T1, T2, T3 and T4 (Figure 2c) were compared with the data of the *Avr3a* ^*GM*^ allele from Avr3a^GM^‐T‐4 (Figure 5a). The threshold values for the induction of spontaneous cell death were shown by a bar. *Avr3a* gene expression was normalized against expression of the potato house‐keeping gene *StMCB75 n* = 4, ± indicates standard derivation.

All six transgenic lines showed an induction of the *Avr3a* gene expression after *P. infestans* infection in comparison with the water controls at 2, 3 and 4 dpi. However, the expression of the *Avr3a* gene differed between the six lines. Recombinant *Avr3a* expression was not detectable for Hermes and was nearly undetectable for line Avr3a^LM^‐T‐6. The expression analysis showed a correlation between *Avr3a* gene expression and resistance improvement. The *Avr3a* gene expression was higher at 4 dpi in the three lines Avr3a^IM^‐T‐2, Avr3a^GM^‐T‐3, Avr3a^GM^‐T‐4, which showed a significant resistance improvement compared with the three lines Avr3a^IM^‐T‐1, Avr3a^LM^‐T‐5, Avr3a^LM^‐T‐6 lacking enhanced resistance. The strongest *Avr3a* expression could be detected for the most resistant line Avr3a^GM^‐T‐4. The absolute expression of the *Avr3a* ^*GM*^ gene in the line Avr3a^GM^‐T‐4 was superior to the other lines at 2, 3 and 4 dpi. *Avr3a* gene expression was nearly undetectable for line Avr3a^LM^‐T‐6, which did not show resistance improvement in any assay (Figure 5a).

### *Avr3a* ^*GM*^ potato revealed an increased threshold for the induction of cell death

The transgenic Russet Burbank lines T1‐T4, which were transformed with the *R3a*‐*Avr3a* ^*KI*^ genes, showed leaf abortion and a differential level of stunted growth in the greenhouse dependent on the *Avr3a* ^*KI*^ expression (Figure 2b,c). Expression analysis by qRT‐PCR showed that even the lowest detected *Avr3a* ^*K*I^ gene expression of 1.8 normalized units in leaves of line T1 triggered spontaneous cell death in the absence of a pathogen.

Since absolute quantification using a standard curve was used for the qRT‐PCR experiments, the *Avr3a* expression of the Hermes line Avr3a^GM^‐T‐4 and the Russet Burbank T1‐T4 lines could be compared. The late blight resistant line Avr3a^GM^‐T‐4 showed a background *Avr3a* expression level of 146.5 relative units in the absence of a pathogen without any detrimental effect for the vegetative growth (Figure 5b). These results demonstrated that the threshold for cell death induction was increased at least 80‐fold in the line Avr3a^GM^‐T‐4 compared with the T1‐T4 lines. Although the decreased sensitivity could also be caused partially by a genotype differences (Hermes vs. Russet Burbank), it is likely that the increased threshold is mainly caused by the presence of the *Avr3a* ^*GM*^ allele.

### Induction of cell death in corn, wheat and soybean by *R3a* and *Avr3a* ^*KI*^

**Figure 6:**
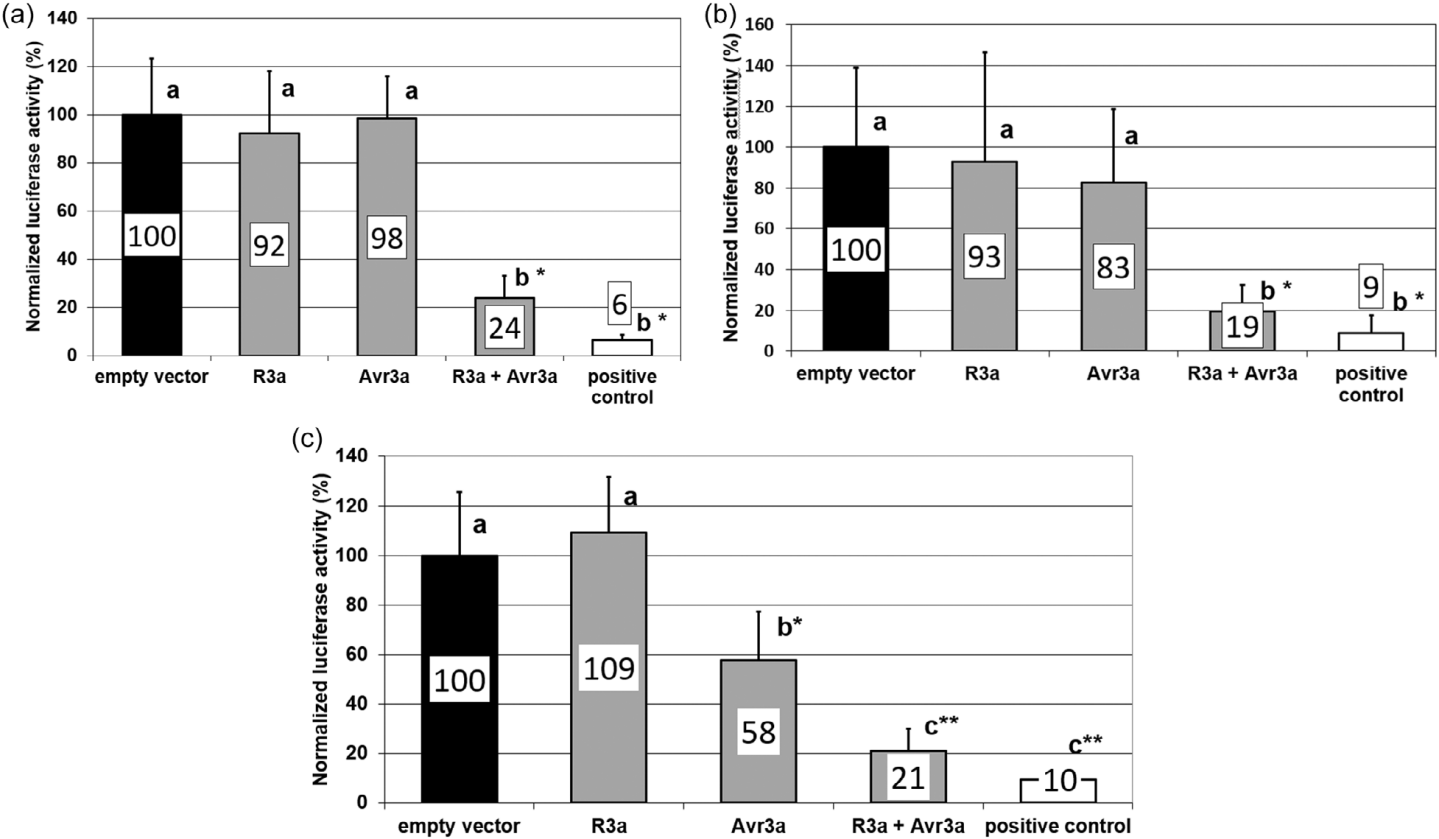
R3a‐Avr3a^KI^ co‐expression triggers cell death in corn, wheat and soybean leaves. The potato *R3a* resistance gene and the *Avr3a* ^*KI*^ gene of *P. infestans* were transiently co‐expressed in leaves of corn, wheat and soybean. The induction of cell death was measured by the activity of two co‐bombarded luciferase reporter genes 16 h after transformation. (a) Co‐expression of the *R3a* and *Avr3a* ^*KI*^ genes in corn leaves triggers cell death in contrast to the single expressed *R3a* or *Avr3a* ^*KI*^ genes. The autoactivated wheat resistance gene *TaMLA1* was used as positive control. Mean of two experiments with six biological replicates of each experiment is shown. (b) Co‐expression of the *R3a* and *Avr3a* ^*KI*^ genes in wheat leaves triggers cell death in contrast to the single expressed *R3a* or *Avr3a* ^*KI*^ genes. An autoactivated wheat resistance gene *TaMLA1* was used as positive control. Mean of three experiments with six biological replicates of each experiment is shown. (c) Co‐expression of the *R3a* and *Avr3a* ^*KI*^ genes in soybean leaves triggers cell death in contrast to the single expressed *R3a* or *Avr3a* ^*KI*^ genes. An autoactivated soybean resistance gene *GmRps1‐k* was used as positive control. Mean of three experiments with six biological replicates of each experiment is shown. Data with different small capitals are different according to ANOVA analysis. *Data of R3a‐Avr3a^KI^ and positive control are statistically different to vector control, *R3a* and *Avr3a* according to a pair‐wise comparison with Tukey test (*t*‐test), *p* < 0.05.

The activity of resistance genes is confined to the members of a plant family, a phenomenon called ‘restricted taxonomic functionality’. In transient assays, the *R3a*‐*Avr3a* ^*KI*^ gene combination was tested for cell death‐inducing activity in other non‐solanaceous crops. After biolistic transformation into leaves of corn, wheat and soybean, a strong induction of cell death was unexpectedly detected as a hallmark of innate immunity (Figure 6). While the *Avr3a* ^*KI*^ gene alone did not cause cell death in maize and wheat, a slight cell death in soybean was already observed for the individually expressed *Avr3a* ^*KI*^ gene. Cell death in soybean was significantly enhanced by co‐expression of the *R3a* and *Avr3a^KI^* genes compared with the expression of *Avr3a^KI^*. Other resistance genes of potato and their matching *Avr* genes triggered cell death in potato leaves but not in leaves of corn and wheat (Figures S5, S6, S7).

The late blight resistance gene *R1* and the *P. infestans Avr1* gene, the late blight resistance gene *Rpi‐blb3* (*R2*) and the *P. infestans Avr2* gene, the virus resistance gene *Rx1* and the potato virus X coat protein gene (*PVX‐CP*) did not induce cell death in wheat leaves after transient co‐expression (Figure S7). The *Avr2* gene under the control of the doubled 35S promoter already induced cell death without the corresponding *Rpi‐blb3* (*R2*)‐gene after overexpression in potato leaves. However, the *Avr2* gene under the control of the synthetic 2xS‐4xD‐NpCABE core promoter triggered cell death in potato only in combination with the corresponding *Rpi‐blb3* gene (Figure S5). The *R1/Avr1* and *Rx1/PVX‐CP* gene combinations did also not induce cell death in corn leaves (Figure S6). This finding showed that the *R3a/Avr3a* gene combination is functional in non‐solanaceous plants and could as well be transferred in combination with a pathogen‐inducible promoter to other crops for the generation of disease resistance.

## Discussion

The combination of an optimized synthetic promoter with a modified *Avr3a* gene allowed the application of the Avr/R concept (de Wit, 1992) to an *R3a* gene expressing potato cultivar. Transgenic potato lines of the cultivar Hermes which expressed the *Avr3a* ^GM^ or *Avr3a* ^IM^ gene under the control of the 2xS‐4xD‐NpCABE_core_ promoter showed enhanced late blight resistance with an inconspicuous growth phenotype.

The potential of synthetic promoters for resistance improvement was already demonstrated in the implementation of the Avr/R concept by Niemeyer *et al*. (2014). Transgenic tobacco plants, which expressed the *p50* avirulence gene of the tobacco mosaic virus (TMV) under control of the elicitor‐ or PAMP‐inducible promoter 2xS‐2xD‐35S_minimal,_ showed resistance to bacterial crown gall in the presence of the TMV resistance gene *N*. The bacteria resistant tobacco plants developed no phenotypes under sterile conditions. However, after transfer to soil, the lines showed spontaneous necrosis of stems and leaves. Although the 2xS‐2xD‐35S_minimal_ promoter allowed the construction of bacteria resistant tobacco plants the application of the *p50*/*N* system was still limited by the development of spontaneous necrosis and the spread of the necrotic reaction to other parts of the plants (Niemeyer *et al*., 2014).

In this report, the synthetic promoters 2xS‐2xD‐35S_minimal_ and 4xW2‐35S_minimal_ showed high background activity in uninfected potato plants and low pathogen induction in comparison with sugar beet and *A. thaliana* (Rushton *et al*., 2002). The high background activity made the promoters unsuitable for the application of the Avr/R concept and indicated that the core promoter has an a hitherto neglected significance for synthetic promoter design and crop‐specific promoter adaption. The best minimal or core promoters for use in synthetic promoters should be inactive in the absence of added cis‐acting elements (Gurr and Rushton, 2005). The technical challenge was solved by the replacement of the 35S minimal promoter by core promoters from *Solanacea* genes which proved to be more suitable. By an analysis of 20 core promoter sequences from different *Solanacea* species, six core promoters of the *NtTGAA*, *Nt5EAS*, *NpCABE*, *StLS1*, *StGst* and *NpATP2* genes were identified with a reduced core promoter activity compared with the 35S minimal promoter. Testing the four core promoters of the *NtTGAA*, *NpCABE*, *StLS1* and *NpATP2* genes with the 2xS‐2xD elements as a proximal region in transgenic potatoes, the synthetic 2xS‐2xD type promoters revealed different background activity, strength and pathogen inducibility. Finally, the 2xS‐2xD‐NpCABE_core_ promoter was selected as the S‐D‐type promoter with the highest inducibility and lowest background activity under non‐disease conditions. The 2xS‐2xD‐NpCABE_core_ promoter was further improved by increasing the number of the cis‐acting D‐element to generate the 2xS‐4xD‐NpCABE_core_ promoter.

The finding that a minimal or core promoter has beside a quantitative as well a qualitative role for the specificity and strength of a synthetic pathogen‐inducible promoter is a new insight. Beside the *cis*‐acting element, the core promoter should be taken too into account as a building block to design a synthetic promoter. The analysis of the architecture of core promoters is the subject of current investigations. The focus of the investigations is to understand the role of the core promoter elements for promoter strength. A genome‐wide analysis of the core promoters of *Arabidopsis thaliana* identified several motifs overrepresented in the core promoter. Beside the TATA box‐element, most of the overrepresented motifs were only present in a subset of the analysed sequences (Molina and Grotewold, 2005). By a comprehensive genome‐wide validation, the core promoter sequences of *A. thaliana*, corn and sorghum were analysed transiently for core promoter strength (Jores *et al*., 2021). The TATA box‐element proved to be the most important element for promoter strength and other known core element as the Inr sequence (Achard *et al*., 2003) or Y patch element showed weaker effects (Jores *et al*., 2021).

The core promoter of a gene is essential for gene expression. Therefore, it is not surprising that this promoter sequence is a target for pathogen mediated defence repression. The phenylalanine ammonia‐lyase (*Pal*) gene of sugar beet is repressed during the early infection stage of *Cercospoa beticola* and the repression is mediated by the core promoter of the *Pal* gene (Schmidt *et al*., 2004). In summary, a study of core promoter architecture with respect to biotic and abiotic stress inducibility and crop‐specific adaptation would be of interest and helpful for a biotechnological application of synthetic promoters.

The co‐expression of the *R3a* gene with the *Avr3a* ^KI^ gene under control of the 2xS‐2xD‐*NpCABE*_core_ promoter in the potato cultivar Russet Burbank resulted in plants with spontaneous cell death symptoms. Depending on the background activity of the synthetic promoter in the transgenic lines, the potato lines showed leaf abortion and growth retardation in adult plants. The basic expression of the *Avr3a* ^KI^ gene was still too high in non‐infected plants. The threshold for cell death induction could be increased by the application of *Avr3a* alleles which trigger less cell death. Bos *et al*. (2009) showed that the amino acid K at Position 80 is important for *R3a* gene activation regardless of the polymorphism at residue 103 in *N. benthamiana*. The published results of tobacco were compared with the data of this study measured in potato leaves. The alleles *Avr3a* ^*KI*^, *Avr3a* ^*KM*^ and *Avr3a* ^*RM*^ triggered a strong cell death in *Nicotiana benthamiana* and potato, whereas the alleles *Avr3a* ^*DM*^, *Avr3a* ^*FM*^, *Avr3a* ^*YM*^ and *Avr3a* ^*WM*^ were not recognized by *R3a* in both plant species. However, other alleles like *Avr3a* ^*HM*^, *Avr3a* ^*CM*^, *Avr3a* ^*NM*^, *Avr3a* ^*PM*^, *Avr3a* ^*SM*^, *Avr3a™*, *Avr3a* ^*AM*^, *Avr3a* ^*IM*^, *Avr3a* ^*LM*^, *Avr3a* ^*MM*^ and *Avr3a* ^*VM*^ induced surprisingly less cell death in potato, and the alleles *Avr3a* ^*PM*^ and *Avr3a* ^*GM*^ revealed a stronger cell death induction than in tobacco. The generation of allelic variants of the *Avr3a* gene finally allowed a balanced expression of the *R3a* and *Avr3a* genes without a detrimental effect but still capable of activating the innate immunity during pathogen infection. The strategy of mutagenized versions of *Avr3a*‐genes having reduced cell death‐inducing activity should be applicable to other combinations of *R*‐genes and *Avr* genes in potato or other crops.

The co‐expression concept with the *R3a*/*Avr3a* gene combination could be transferred as well directly to several other crops. Surprisingly, the *R3a* gene and the corresponding *Avr3a* gene are functional in soybean and monocots as corn and wheat. The biolistic assays showed that the co‐expression of the *R3a*/*Avr3a* genes resulted in cell death in leaves of soybean, corn and wheat. This functionality cannot be taken for granted since the co‐expression of other potato *R*/*Avr* gene combinations failed to induce cell death in corn and wheat.

The functionality of a single *R*‐gene is usually restricted to a plant family or even to a species. This phenomenon is named ‘restricted taxonomic functionality’. Heterologous expression of NB‐LRR type *R*‐genes in a taxonomically distinct family triggers either no response or inappropriate immunity, suggesting that the regulatory or signalling components associated with NB‐LRR protein‐based resistance are family‐specific (Brutus *et al*., 2010). Resistance gene transfer between species may be limited not only by divergence of signalling effector molecules and pathogen avirulence ligands, but potentially also by more fundamental gene expression and transcript processing limitations (Ayliffe and Lagudah, 2004).

A break of the restricted taxonomic functionality was reported by the transfer of a pair of NB‐LRR‐type *R*‐genes, *RPS4* and *RRS1* (Narusaka *et al*., 2013) from a *Brassicaceae* plant (*Arabidopsis thaliana*) into two *Solanaceae* plants (*Nicotiana benthamiana*, *Solanum lycopersicum*). An interfamily transfer from a *Solanaceae* plant (*Solanum lycopersicum*) to a *Brassicaceae* plant (*Arabidopsis thaliana*) was described for the RLP‐type R‐protein Ve1 (Fradin *et al*., 2011). However, although Ve1 is considered to encode a race‐specific resistance protein, several observations support the hypothesis that Ve1 is an ancient pathogen receptor with traits of typical PRRs (Fradin *et al*., 2011). It is known that PRRs can be transferred across species boundaries, as exemplified by the transfer of the PRRs *EF‐Tu* and *FLS2* genes from *A. thaliana* to *N. benthamiana* and *S. lycopersicum*. Until now, only alleles of certain single NB‐LRR *R*‐genes, as well as one two‐component NB‐LRR resistance gene pair were found to be suitable for an interfamily transfer from the family of *Poaceae* or *Gramineae* into the plant families of *Solanaceae* and *Brassicaceae*. The barley powdery mildew resistance gene *Mla1* triggers cell death in *A. thaliana* after co‐expression with the corresponding avirulence gene *AVR*_*a1*_ (Lu *et al*., 2016). Its wheat orthologs *Sr33* and *Sr50* trigger cell death when overexpressed in *N. benthamiana* leaves (Cesari *et al*., 2016). The wheat powdery mildew resistance genes *Pm3a* and *Pm3f* trigger cell death after co‐expression with the corresponding avirulence gene *AvrPm3* ^*a2/f2*^ in *N. benthamiana* (Bourras *et al*., 2015). Generally, the distant relationship between monocot plants and dicot plants makes it difficult to predict whether a resistance gene originating from the one group can trigger an immune response in the other group.

The transfer of the modified *Avr3a* alleles of *Phytophthora infestans* under the control of a synthetic pathogen‐inducible promoter resulted in GM potato lines with enhanced late blight resistance. The best line Avr3a^GM^‐T‐4 showed a reduced sporangia production of 60% in the DLA. The maximal effect might be even higher since only eight randomly selected transgenic lines of each construct were analysed. The number of sporangia produced at the end of an infection cycle is very important for the epidemiology of late blight. Sporangia can be transported over long distances by wind and infection of leaves happens either by germinating sporangia or the release of six to eight zoospores from one sporangium. Under favourite conditions, one disease cycle of *P. infestans* from infection until sporulation last only 4 days and several infections cycles happen during a potato growing season. Starting from one infected plant billions of spores could be produced in the field (Kamoun *et al*., 2015). A reduction of 60% sporangia at each cycle would have a significant effect to limit the spread of the disease. The synthetic S‐D type promoters are induced by several fungal pathogens in different crops (Stahl, unpublished results). Additional experiments must show if the Avr3a^GM^ lines possess not only a late blight but also a multiple fungal resistance.

## Experimental procedures

### Plant and *P. infestans* cultivation

Potatoes of the cultivars Hermes, Russet Burbank and the derived transgenic lines were grown in 3 L pods for biolistic transformation assays and in 5 L pods for detached leaf assays in the greenhouse at 22°C day and 16°C night temperature. Corn plants of the cultivar Simao (KWS SAAT SE) were grown in 1 L pods at 24°C day and 18°C night temperature in the greenhouse.

Soybean plants of the cultivar Merlin (Saatzucht Linz) were grown in 40 well plates on steamed soil in a Percival growth chamber for biolistic transformation assays. Seeds were initially surface sterilization with 3% hydrogen peroxide, pre‐germinated for 3 days on filter paper in the dark at room temperature and then transferred to the 40 well plates. Cultivation took place at 25°C and 15 h light with a weekly application of liquid fertilizer.

Wheat plants of the spring wheat cultivar KWS Taifun (KWS SAAT SE) were grown in 40 well plates on greenhouse soil in the greenhouse. Cultivation took place at 18°C with 16 h day length and 16°C at night.

The late blight isolate Gross‐Luesewitz was cultivated for maintenance and infection assays on tuber slices of surface sterilized potatoes in a plastic box. After 1 week of cultivation the mycelium with the sporangia was carefully harvested from the potato slices and incubated in water at 12°C for 4 h to induce the release of zoospores. The zoospores were used to reinfect fresh potato slices or to infect potato leaves after determination of zoospore concentration.

### Detached leaf assay

Transgenic potato lines and non‐transgenic control varieties were grown from *in vitro* plants in 5 L plant pots for resistance assays in the greenhouse. After 6–8 weeks one leaf with nine well‐developed leaflets was chosen from each plant and was placed in a translucent plastic box on a moist layer of Grodan stone wool. Four leaves from different lines were placed in each plastic box. In total, eight leaves from eight plants were analysed for each line in one assay in a randomized design. Five leaflets from each leaf were inoculated. The top leaflet was inoculated at two sites with two 10 μL droplets and the second to fifth leaflets were inoculated at one site with one 10 μL droplet of a *P. infestans* zoospore suspension with a concentration of 10^4^ zoospores × mL^−1^. After 1 day the transparent lids of the plastic boxes were slightly opened to assure some air circulation in the boxes. Boxes were incubated at 18°C in day/night rhythm in the greenhouse. Quantification of sporangia was performed at 6 dpi. Infected leaves were incubated for 2 h in Falcon tubes with 5 mL H_2_O on a roll mixer to wash off sporangia from the leaves. The number of sporangia was counted under the microscope in a Thoma counting chamber.

### Late blight field assays

Race specificity of the late blight isolate Gross‐Lueswitz was assessed in a field trial using the *S. demissum* introgressions R1 to R11 and a line R0 without qualitative resistance (Mastenbroek, 1952). Plots of 12 plants were planted from *in vitro* grown cutlings after cultivation of 21 days under greenhouse conditions. The plot design was consisting of two plots next to each other, each double plot row was surrounded of a spreader row of the susceptible cultivar Nicola. After a total growth period of 6 weeks inoculation of the spreader row were conducted multiple times in the evening hours by spray inoculation of a sporangia suspension of 20 000 sporangia/mL.

Disease symptoms were scored for 3 weeks on a plot basis on scale from 0 to 100. Starting with 0–10 for individual symptoms and 10–100 assessing plot foliage destruction. For further analysis, AUDPC was calculated and significance was assessed using two‐way ANOVA.

### *Cercospora beticola* infection of sugar beet

The infection of transgenic sugar beets with *C. beticola* was done in an *in vitro* assay as described (Schmidt *et al*., 2008).

### Cloning of core promoter constructs

The cloning of the 20 core promoter sequences was done by hybridization of two 41–46‐bp large overlapping oligonucleotides and a fill‐in reaction of the single stranded 3′‐ends by the Klenow‐fragment. The TATA box of each core promoter was located at Position 19–24 of the 67–75‐bp large DNA fragments. The forward primers had a *PstI*, and the reverse primers a *XhoI* recognition sequence at the 5′‐end, which was used for the replacement of the 35S minimal promoter of the vector MS23‐luc‐m3 by the core promoters to generate the core‐luc‐m3 vectors.

The core promoter sequences of the chlorophyll a/b‐binding protein (*NpCABE*, Accession X12512) and the beta subunit of the mitochondrial ATP synthase gene (*NpATP2*, Accession X02868) from *Nicotiana plumbaginifolia* were 5′‐AACTGCAGTAAAGCCATTTATATACACTTA GTGCAAAGCCCATGAAACTCAAGCCTCAACTCGAGTT‐3 and 5′‐AACTGCAGTGGGCTC TTGATATATCAGCTCTTCACAAACCCTAGCAGTCTCTTTCTCTCCTCTCGAGTT‐3, respectively.

From *Nicotiana tabacum*, the selected core promoter sequence of the TGA transcription factor gene (NtTGAA, Accession M62855) was 5′‐AACTGCAGTAAAGCCATTTATATACA CTTAGTGCA AAGCCCATGAAACTCAAGCCTCAACTCGAGTT‐3′. The core promoter of the 5‐epi‐aristolochene synthase gene (*Nt5EAS*, Accession L04680) was 5′‐AACTGCAGCAG TTTAATGTACATTACATATAGGTTGCGGAAAAGTATATATATGCTCAAGACTCGAGTT‐3′. The core promoter of the small subunit of the ribulose bisphosphate carboxylase gene (*NtRBS*, X02353) was 5′‐ACTGCAGCCTTATCATTATATATAGGGTGGTGGGCAACTATGCAAGACC ATATTGGACTCGAGTT‐3′. The core promoter of the endochitinase gene (*NtCHI2*, Accession X64519) was 5′‐AACTGCAGCTTCCTTACCAATAAATACCTTGCACTTCGCCACTTTACTA CTACATC AAAAATCTCGAGTT‐3′. The core promoter of the nitrate reductase gene (NtNIA1, Accession X14058) was 5′‐AACTGCAGCGTAACGTTTCTATATAAGGCCACCCCACGCAT TCACAAACTTCGTTCGAAAACTCGAGTT‐3′ and the core promoter of a 1,3‐beta glucanase gene (*NtE13E*, Accession X53600) was 5′‐AACTGCAGTATCTCAATATAAATAGCTCGTT GTTCATCTTAA TTCTCCC AACAAGTCTTCTCGAGTT‐3′.

The core promoter sequences from genes of *Solanum tuberosum* are listed below. The core promoter of the light‐induced LS1 gene (*StLS1*, Accession X04753) was 5′‐AACTGCAG ACCATGCAAAGTGAAAATAAATAATTCATACTAAGTAGTGAGAGCAAAGAAGAAAAAAGCTCGAGTT‐3′. The core promoter of the glutathione S‐transferase gene (*StGst*, Accession J03679) was 5′‐AACTGCAGAGTCAAATATAATTTTATATTAGAATAATTGAATAGTCAAAC AAGAAACTTTAACGAGT‐3. The core promoters of the proteinase inhibitor genes *StIP2K* and *StCID* (Accession M29965 and M17108) were 5′‐AACTGCAGCACTCGTTTGCTATAAATAG GTGGAGGAGGACAGACACTCTTCACCCCAAACTCGAGTT‐3′ and 5′‐AACTGCAGACCTC TGCCTATAAATTTAAGTGATGCACTCATACAAATTCACTCAATTCCTCTCGAGTT‐3′. The core promoter of the p‐coumaroyl‐CoA‐ligase gene (*St4CL*, Accession AF150686) was 5′‐AACTGC AGGTTTTTTTTTCTCATATATATATTCATCAATCTTTGCACATTCATCTTCAGGA CACCCTCGAGTT‐3′. The core promoters of phenylalanine ammonia‐lyase genes *StPAL1* and *StPAL2* (Accession X63103 and X63104) were 5′‐AACTGCAGTTTTTTATTATCAT ATATCTATATATACAAATAAACTCTAAGCATTTTTCTTCACCTCGAGTT‐3 and 5′‐AACTGC AGCACAACATTTTTTTTTATATATATACAAATAAACTCTAAGCATTTTTCTTCACCTCGAGTT‐3′. The core promoter of a metallo carboxypeptidase inhibitor gene (*StMCPI*, Accession AJ242665) was 5′‐AACTGCAGACTAAGGGGCCTATAAATTGGACCCTTCTCAAAGAAA AATAAAATCACCACTCAACTCGAGTT‐3′. The core promoters of the small and the large subunit of the ADP‐glucose pyrophosphorylase genes (*StADP*, Accession X96771; *StADPGP*, Accesion X75017) were 5′‐AACTGCAGGACAAAATCAGGTCTATAAAGTTACCCTTGATAT CAGTATTATAAAACTAAAAATCTCAGCTCGAGTT‐3′ and 5′‐AACTGCAGGGAAATGAGTA TAAATAGAAAGATAGCAAGGTTTCTCGTGAGAGTTCACAAGCCAATAAACTCGAGTT‐3′. The core promoter of the 1‐aminocyclopropane‐1‐carboxylate synthase (*StACS*, Accesion Z27235) was 5′‐AAC TGCAGTAAACTACTTTCTATATAAGTTGCTGCTCTTTGCCAAAAAAA GTTCATATTCA AAC ACTCTCGAGTT‐3′. The core promoter of a cold‐stress induced gene (*StC17*, Accession U69633) was 5′‐AACTGCAGGTTTGTAATTGCTATATAAAGCGCACC AAGTTTCATATTTC TACCACATCCAATTCAAACTCGAGTT‐3′.

The enhancer of the 35S promoter was inserted as a *HindIII‐Eco32I* fragment into the *HindIII‐PdiI* sites upstream of the core promoter sequences NtTGAA, Nt5EAS, NpCABE, StLS1, StGST and NpATP2 of the core‐luc‐m3 vectors to generate the enhancer–core promoter constructs.

### Cloning of synthetic promoter reporter gene constructs

The cis‐acting elements 2xS‐2xD and 4xW2 were inserted as *SpeI‐XbaI* fragments upstream of the 35S minimal promoter and the luciferase gene of the vector MS23‐luc‐m3 to generate the vectors 2xS‐2xD‐luc and 4xW2‐luc. The expression cassette of the synthetic promoters 2xS‐2xD‐35S_minimal_ and 4xW2‐35S_minimal_ in combination with the *P. pyralis* luciferase reporter gene was inserted into the binary vector pGPTV‐Kan (Becker *et al*., 1992) for plant transformation.

The 35S minimal promoter of the vector 2xS‐2xD‐luc was replaced by *SpeI‐XbaI* fragments with the core promoter sequences of the NtTGAA, NpCABE, StLS1 and NpATP2 genes generating the vectors 2xS‐2xD‐NtTGAA_core_‐luc, 2xS‐2xD‐NpCABE_core_‐luc, 2xS‐2xD‐StLS1_core_‐luc and 2xS‐2xD‐NpATP2_core_‐luc. The 2xS‐2xD cis‐acting elements of the vector 2xS‐2xD‐NpCABE_core_‐luc were replaced as *SpeI‐XbaI* fragments by the 4xS‐2xD and 2xS‐4xD elements to generate the vectors 4xS‐2xD‐NpCABE_core_‐luc and 2xS‐4xD‐NpCABE_core_‐luc. The expression cassettes of these reporter gene vectors were inserted into the binary vector pGPTV‐Kan for plant transformation.

### Cloning of Avr3a constructs

The avirulent *Avr3a* ^*K80I103*^ allele was amplified and subcloned without the signal sequence in a two‐step process. First, the full‐length coding region of the *Avr3a* ^*K80I103*^ allele was amplified with the primers Avr3a‐for (5′‐ATGCGTCTGGCAATTATGCT‐3′) and Avr3a‐rev (5′‐CTAATATCCAGTGAGCCCCA‐3′) from *P. infestans* and subcloned into the vector pGEM‐T Easy (Promega). Second, the *Avr3a* ^*K80I103*^ allele without signal sequence was amplified with the primers Avr3a‐forII (5′‐AACTCGAGATGGACCAAACCAAGGTCCTGGTG‐3′) and Avr3a‐rev (5′‐CTAATATCCAGTGAGCCCCA‐3′) using the subcloned full‐length *Avr3a* ^*K80I103*^ fragment as a template. The *Avr3a* ^*KI*^ allele was inserted downstream of the 35S promoter with a doubled CaMV enhancer of the vector pCaMV‐2 to generate the expression vector p70S‐Avr3a^KI^. The *Avr3a* ^*KI*^ allele of p70S‐Avr3a^KI^ was replaced by a synthesized virulent *Avr3a* ^*EM*^ allele to generate the plasmid p70S‐Avr3a^EM^. Starting from the construct p70S‐Avr3a^EM^ the amino acid E at Position 80 was substituted by all natural amino acids except for W. For each substitution a 346 large DNA fragment was synthesized (Eurofins, Germany) and inserted as *XhoI‐BglII* fragment into p70S‐Avr3a^EM^ to generate the alleles *Avr3a* ^*DM*^, *Avr3a* ^*FM*^, *Avr3a* ^*YM*^, *Avr3a* ^*HM*^, *Avr3a* ^*KM*^, *Avr3a* ^*RM*^, *Avr3a* ^*CM*^, *Avr3a* ^*NM*^, *Avr3a* ^*PM*^, *Avr3a* ^*QM*^, *Avr3a* ^*SM*^ *Avr3a* ^*TM*^, *Avr3a* ^*AM*^, *Avr3a* ^*GM*^, *Avr3a* ^*IM*^, *Avr3a* ^*LM*^, *Avr3a* ^*MM*^ and *Avr3a* ^*VM*^.

For the co‐expression of the genes *R3a* and *Avr3a* ^*K*I^ in Russet Burbank, the binary vector pBIN‐*R3a*::2xS‐4xD‐NpCABE_core_‐*Avr3a* ^*K*I^ was constructed. The luciferase gene of 2xS‐4xD‐NpCABE_core_‐luc was replaced by *Avr3a* ^*K*I^ and the *Avr3a* expression cassette was inserted into the vector pBIN‐*R3a* (Uni Wageningen). For the transformation of Hermes with the alleles *Avr3a* ^*IM*^, *Avr3a* ^*GM*^ and *Avr3a* ^*LM*^, the luc gene of 2xS‐4xD‐NpCABE_core_‐luc was replaced by the alleles *Avr3a* ^*IM*^, *Avr3a* ^*GM*^ and *Avr3a* ^*LM*^ and the expression cassettes inserted into pGPTV‐Kan.

### Cloning of autoactive resistance genes

The rust resistance gene *GmRps1‐k* and the powdery mildew resistance gene *TaMLA1* were autoactivated to be used as positive controls in the cell death assays. The autoactivated R‐genes were cloned into the vector pCaMV under the control of the 35S promoter with doubled enhancer.

### Transient biolistic experiments

Transient biolistic experiments were carried out using the PDS‐1000/He system (Bio‐Rad) as described (Schmidt *et al*., 2004) on 9‐cm petri‐dishes with a distance between the stopping screen and the leaf sample of 12 cm in a vacuum of 0.0965 MPa (28.5 inches) Hg. The protocol was modified for corn, potato, soybean and wheat as followed:

#### Corn

Four to five leaf segments of 1.5 cm length were cut from either the second or third leaf of 12–24‐day‐old corn plants with a scissor. For an experiment only, the leaves of an identical developmental stage were used. Four or six leaf segments derived from different plants were put on a MS + 0.4 M mannitol agar plate for bombardment using a pressure of 9.3 MPa (1350 psi).

#### Potato

Leaf discs (1 cm diameter) from leaves of 6–8 weeks old greenhouse plants of the cultivar Hermes were cut with a cork borer, mingled and put on MS + 0.4 M mannitol agar plates for bombardment. Eight leaf discs on each agar plate were bombarded with the DNA coated micro‐carriers, using a pressure of 9.3 MPa (1350 psi).

#### Soybean

Leaf discs (1 cm diameter) from the first, second or third leaf were cut with a cork borer, mixed and put on MS + 0.4 M mannitol agar plates for bombardment. Eight leaf discs on each agar plate were bombarded with the DNA coated micro‐carriers, using a pressure of 10.68 MPa (1550 psi).

#### Wheat

Four to five leaf segments of 1.5 cm length were cut from either the first, second or third leaf of 17–30‐day‐old wheat plants. For an experiment, only the leaves of an identical developmental stage were used. Eight leaf segments derived from different plants were put on a MS + 0.4 M mannitol agar plate for bombardment and bombarded using a pressure of 10.68 MPa (1550 psi).

For the analysis of the core promoter sequences, a fusion of the core promoter and the luciferase gene (*Luc*) of *Photinus p*yralis were mixed with the internal standard p70SRuc, a fusion between the doubled 35S‐promoter, and the coding sequence of the *Renilla reniformis* luciferase in a ratio of 1:1 (w:w). DNA was precipitated onto gold micro‐carriers (Au Typ 200‐03; Heraeus, Hanau, Germany). After bombardment, leaf samples were incubated for 16 h at 25°C in the light. Activity of both luciferases were quantified using a dual luciferase assay (Promega) as described (Schmidt *et al*., 2004). Relative reporter gene activity was calculated as follow:

$$\;\left[\text{Photinus value}\;\left(\text{reporter gene construct}\right)–\;\text{Photinus value}\;\left(\text{without}\;\mathrm{DNA}\right)/\;\text{Renilla value}\;\left(\text{normalization construct}\right)–\;\;\text{Renilla value}\;\left(\text{without}\;\mathrm{DNA}\right)\right]\times 100.$$

The given mean was the average of 3–10 replications.

For the measurement of cell death induced by the *Avr3a* alleles and *Avr3* ^*KI*^ in combination with the *R3a* gene, the reporter gene constructs were precipitated onto two charges of gold micro‐carriers. The *Avr3a* construct and the *R3a* construct were precipitated together with p70S*Luc*, a fusion between the doubled 35S‐promoter, and the coding sequence of the *Photinus pyralis* luciferase onto one gold charge. The internal standard p70S*Ruc* was precipitated onto the second gold charge. The micro‐carriers with the *Avr3a*, *R3a*, p70S*Luc* constructs and p70S*Ruc* were mixed in a ratio of 1:4 (w:w). The separation of the two reporter genes and the application of excess gold particles with the p70S*Ruc* construct allowed a normalization of the reporter gene data.

### Plant transformation

*Agrobacterium tumefaciens* mediated transformation of potato internodes were performed according to a slightly modified protocol (Pel *et al*., 2009) by using the antibiotic Kanamycin. Internodes of the potato variety Hermes and Russet Burbank were transformed with bacteria containing derivates of the binary vector pGPTV‐Kan (Becker *et al*., 1992). Kanamycin‐resistant shoots were placed on rooting medium and tested for construct integration via diagnostic PCR with the primer Bo2299 (5′‐GTGGAGAGGCTATTCGGTA‐3′) and Bo2300 (5′‐CCACCATGATATTCGGCA AG‐3′) to amplify a 553‐bp large fragment of the nptII gene. Sugar beet transformation was done as described (Schmidt *et al*., 2008).

### *Avr3a* gene expression analysis

For the time course analysis of the *Avr3a* gene expression, the potato leaves were inoculated with a zoospore suspension of *P. infestans* as described for the DLA. Deviating from the DLA, each leaflet was inoculated with 10 drops of zoospores. RNA was extracted from inoculated and control leaves 0, 1, 2, 3 and 4 dpi using the RNAeasy Kit (Qiagen). For RT‐PCR, the first‐strand cDNA was prepared using RevertAid First Strand cDNA Synthesis Kit (Thermo Scientific). Real‐time RT‐PCR (qRT‐PCR) was performed on a ViiA7 Cycler with SYBR Green Supermix (Life technologies), following a two‐step protocol: 95°C for 10 min, 40 cycles of denaturation at 95°C for 15 s and annealing/extension at 60°C for 1 min. Expression of the recombinant *Avr3a* gene of six transgenic lines and the cultivar Hermes was measured by qRT‐PCR analysis using the primers S3169 (5′‐AATCAGATCTACAATAGCTACATG ATGCA‐3) and S3170 (5′‐CCTTATCTGGGAACTACTCACACA‐3′). *Avr3a* gene expression was normalized against the expression of the potato gene *StMCB75* using the primers S1494 (5′‐TAAGAAAGGCAAGGCTGCAT‐3′) and S1495 (5′‐TCCCTTGGGCAA GTTAACAG‐3′).

### Reporter gene analysis

For quantitative luciferase reporter gene, assays of transgenic sugar beets and potatoes specific amounts of leaf tissue were homogenized in four volumes of cell culture lysis reagent (Promega), and luciferase activity was determined with the luciferase assay reagent (Promega) as described (Schmidt *et al*., 2008).

Histochemical localization of luciferase activity of *P. infestans* inoculated potato leaves were done by spraying detached leaves with a solution of 5% (v/v) dimethyl sulfoxide (DMSO) and 100 μM luciferin. The plants were photographed under white light, and luciferase activity was then detected in the dark using a MicroMax digital CCD camera (Visitron Systems, Germany).

## Author Contributions

Friedrich Kauder contributed to the design of synthetic promoter project, generation and analysis of transgenic reporter gene plants. Klaus Schmidt contributed to the design of synthetic promoter project and core promoter work, implementation of core promoter work, detection of local promoter activity. Gabor Gyetvai contributed to the generation of transgenic R3a/Avr3a plants, analysis of the pathotype of the *P. infestans* isolate. Daniel Stirnweis contributed to the identification of R3a‐Avr3a recognition in different crops. Tobias Haehre contributed to the detached leaf assays. Kai Prenzler contributed to the generation of transgenic R3a/Avr3a plants. Anja Maeser contributed to the expression analysis of *Avr3a* gene by qRT‐PCR. Christine Klapprodt contributed to the cloning and biolistic analysis of *Avr3a* alleles. Florian Tiller contributed to the biolistic analysis of *R‐Avr* alleles. Jens Lübeck contributed to the general project design. Dietmar J. Stahl contributed to the general project design and *Avr3a* allele work, writing of the manuscript.

## Supporting information


**Figure S1** The 2xS‐2xD‐35S_minimal_ promoter showed low background activity and high inducibility after *Cercospora beticola* infection of transgenic sugar beet lines.

**Figure S2** Analysis of the race composition of the *P. infestans* strain Gross‐Luesewitz in a field trial.

**Figure S3** *R3a‐Avr3a* ^*KI*^ co‐expression did not harm the development of young Russet Burbank plants.

**Figure S4** Phenotype of transgenic Baltica lines transformed with 2xS‐4xD‐NpCABEcore‐*Avr3a* ^*K*I^.

**Figure S5** Potato resistance genes *R1*, *Rpi‐blb3* and *Rx* trigger cell death in potato leaves after co‐expression with the corresponding *Avr* genes.

**Figure S6** Potato resistance genes *R1*, *Rpi‐blb3* and *Rx* are not functional in corn.

**Figure S7** Potato resistance genes *R1*, *Rpi‐blb3* and *Rx* are not functional in wheat.

**Table S1** DNA sequence of the D‐box, S‐box, W2‐box and 35S minimal promoter.

**Table S2** Phenotype and late blight resistance of transgenic Baltica lines transformed with 2xS‐4xD‐NpCABE_core_::Avr3a^KI^.

## Data Availability

The data that support the findings of this study are available on request from the corresponding author. The data are not publicly available due to privacy or ethical restrictions.
