# Supplementary figures and images for: Expression of a modified Avr3a gene under the control of a synthetic pathogen‐inducible promoter leads to Phytophthora infestans resistance in potato
Source: Plant Biotechnol J. 2025 Mar 9;23(5):1683–701. doi: 10.1111/pbi.14615 (PMC12018830; doi:10.1111/pbi.14615)

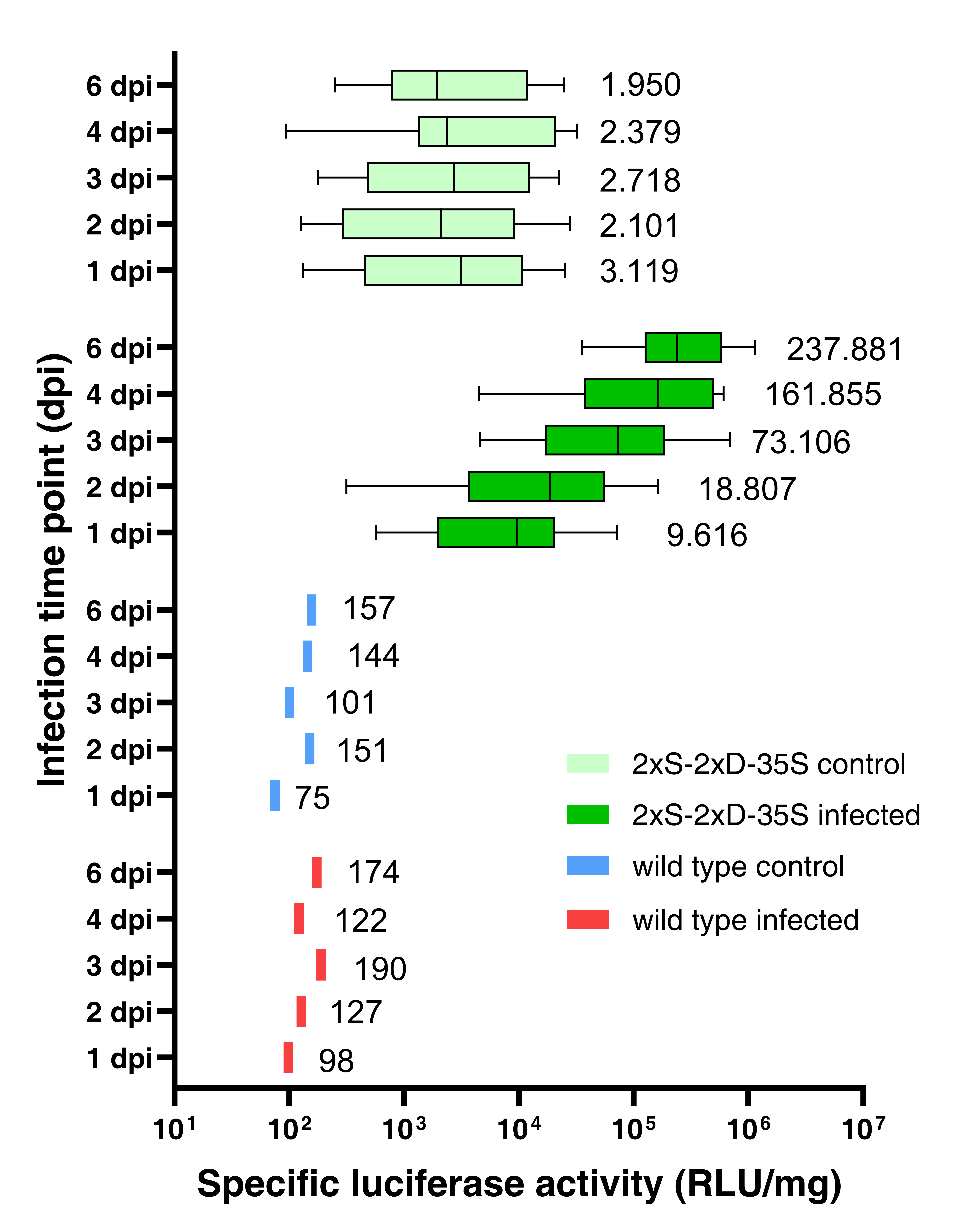

Supplement: Supplementary file 1 — Figure S1 The 2xS‐2xD‐35Sminimal promoter showed low background activity and high inducibility after Cercospora beticola infection of transgenic sugar beet lines. [file PBI-23-1683-s001.png]

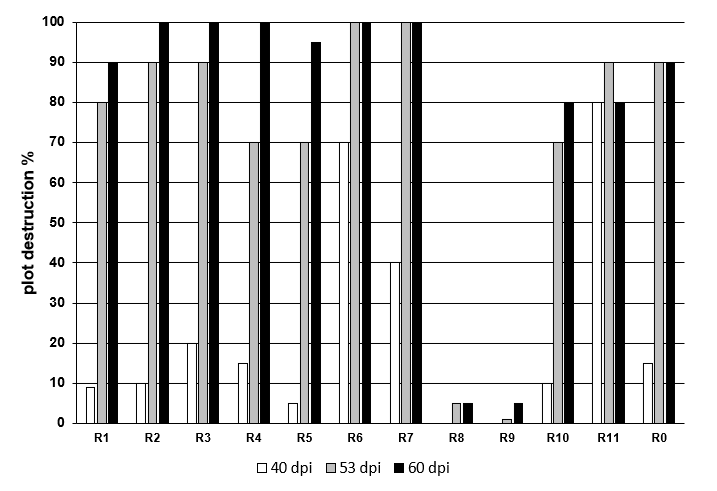

Supplement: Supplementary file 2 — Figure S2 Analysis of the race composition of the P. infestans strain Gross‐Luesewitz in a field trial. [file PBI-23-1683-s002.png]

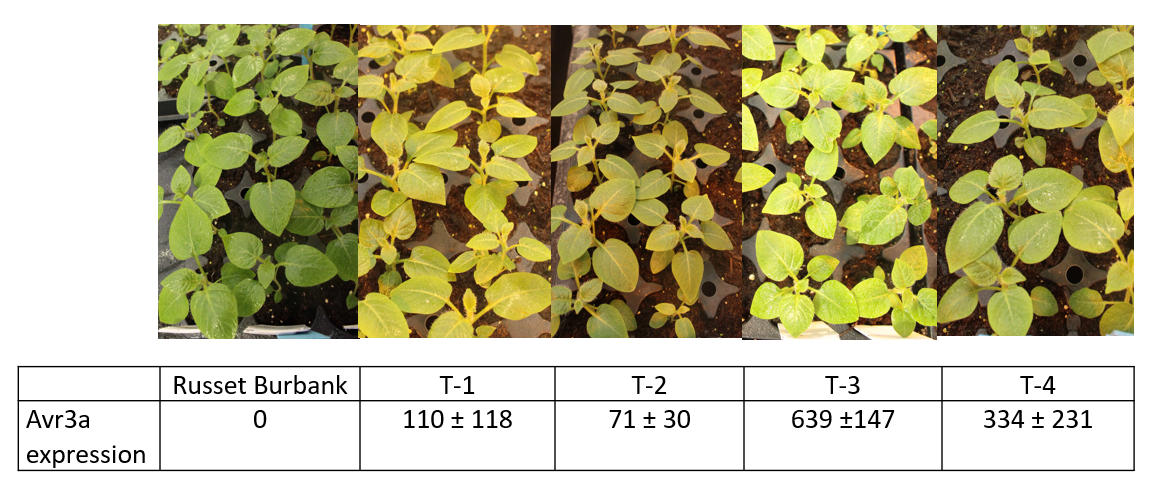

Supplement: Supplementary file 3 — Figure S3 R3a‐Avr3a KI co‐expression did not harm the development of young Russet Burbank plants. [file PBI-23-1683-s009.png]

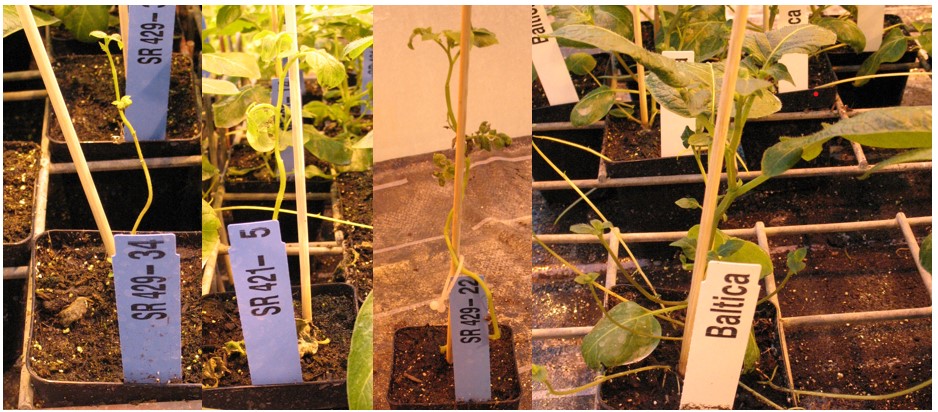

Supplement: Supplementary file 4 — Figure S4 Phenotype of transgenic Baltica lines transformed with 2xS‐4xD‐NpCABEcore‐Avr3a KI. [file PBI-23-1683-s005.jpg]

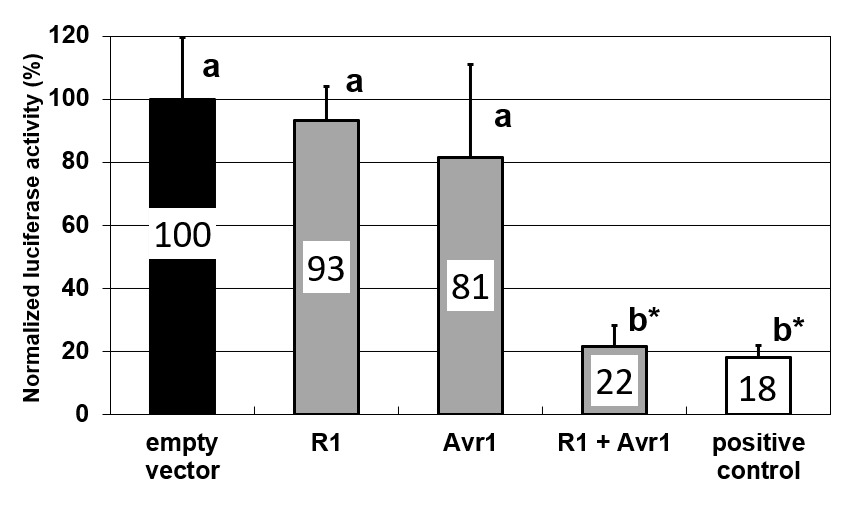

Supplement: Supplementary file 5 — Figure S5 Potato resistance genes R1, Rpi‐blb3 and Rx trigger cell death in potato leaves after co‐expression with the corresponding Avr genes. [file PBI-23-1683-s003.zip › pbi14615-sup-0005-FigureS5a.png]

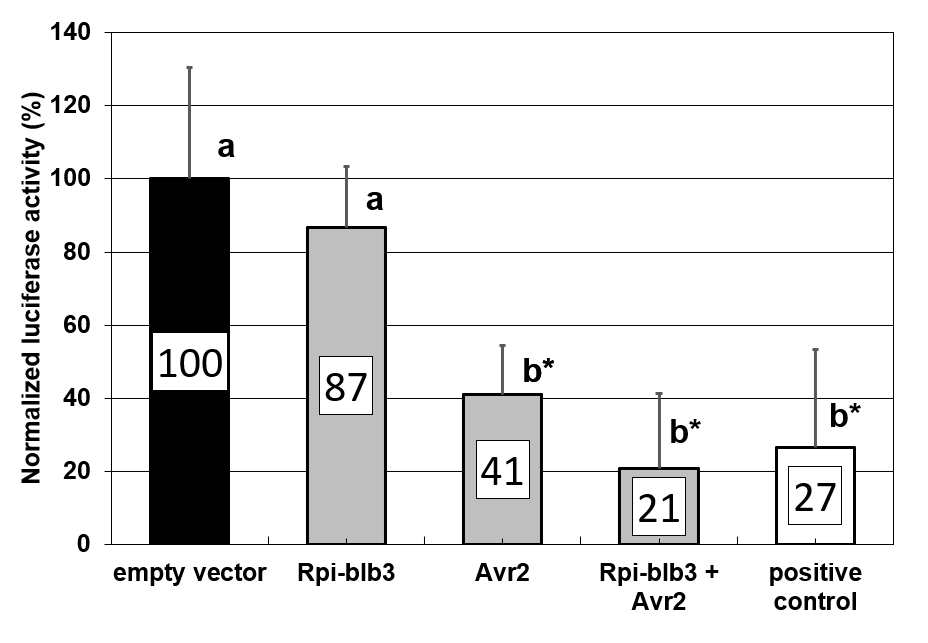

Supplement: Supplementary file 5 — Figure S5 Potato resistance genes R1, Rpi‐blb3 and Rx trigger cell death in potato leaves after co‐expression with the corresponding Avr genes. [file PBI-23-1683-s003.zip › pbi14615-sup-0005-FigureS5b.png]

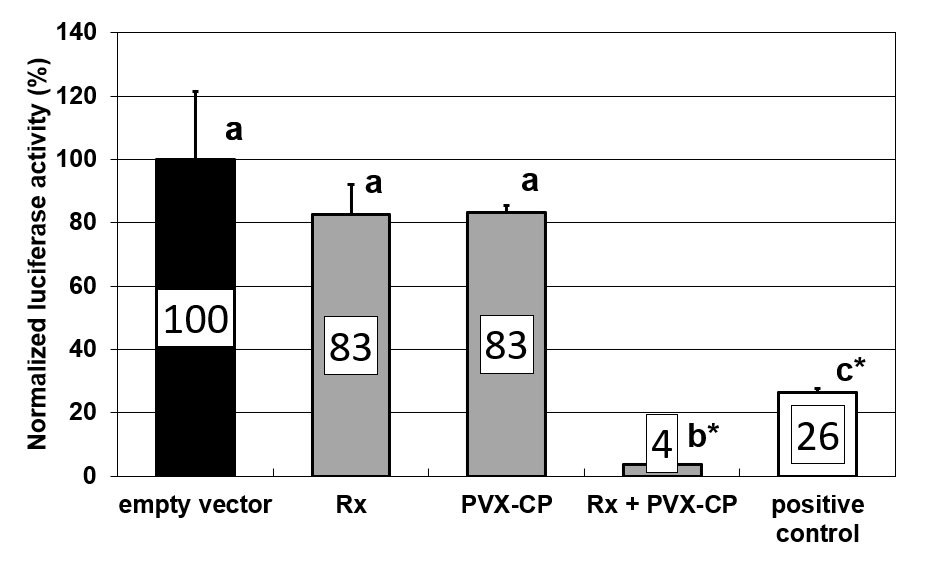

Supplement: Supplementary file 5 — Figure S5 Potato resistance genes R1, Rpi‐blb3 and Rx trigger cell death in potato leaves after co‐expression with the corresponding Avr genes. [file PBI-23-1683-s003.zip › pbi14615-sup-0005-FigureS5c.png]

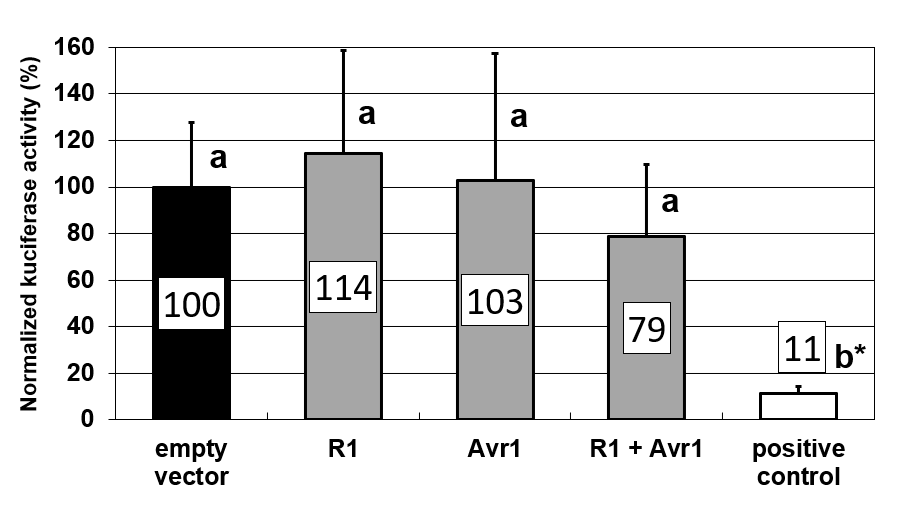

Supplement: Supplementary file 6 — Figure S6 Potato resistance genes R1, Rpi‐blb3 and Rx are not functional in corn. [file PBI-23-1683-s004.zip › pbi14615-sup-0006-FigureS6a.png]

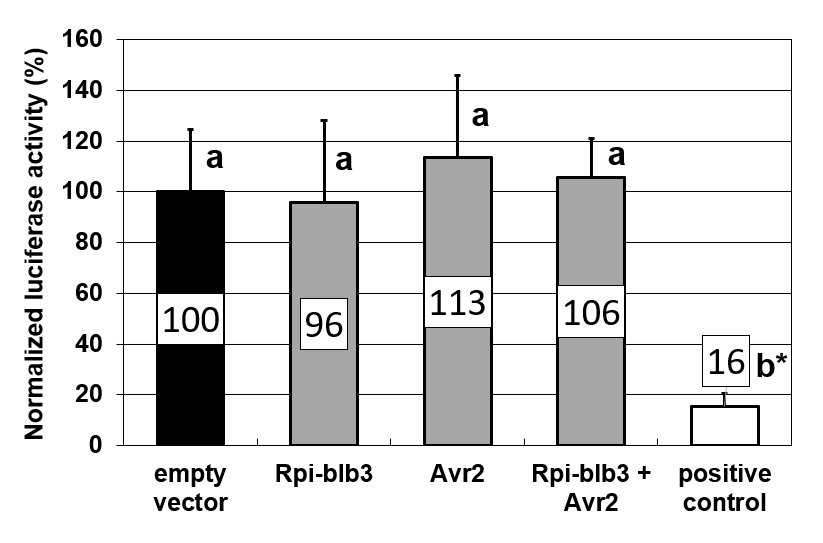

Supplement: Supplementary file 6 — Figure S6 Potato resistance genes R1, Rpi‐blb3 and Rx are not functional in corn. [file PBI-23-1683-s004.zip › pbi14615-sup-0006-FigureS6b.png]

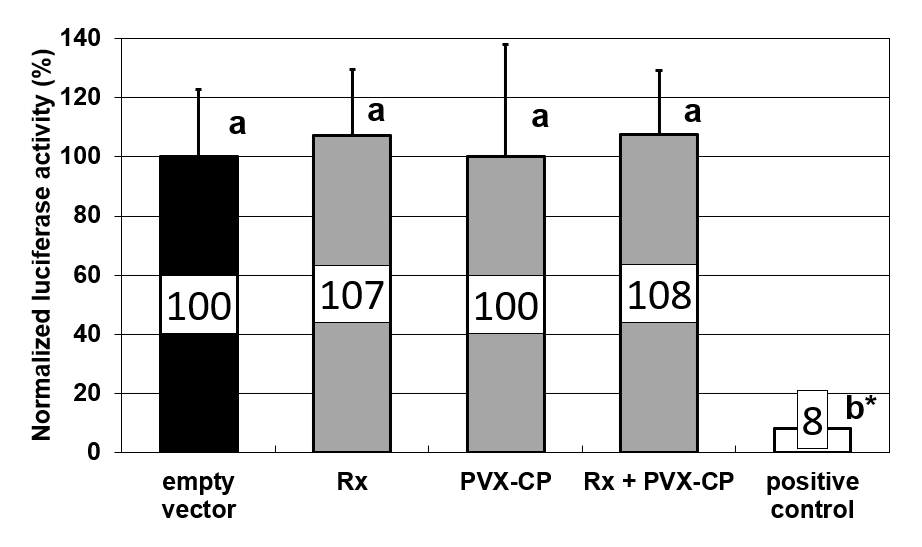

Supplement: Supplementary file 6 — Figure S6 Potato resistance genes R1, Rpi‐blb3 and Rx are not functional in corn. [file PBI-23-1683-s004.zip › pbi14615-sup-0006-FigureS6c.png]

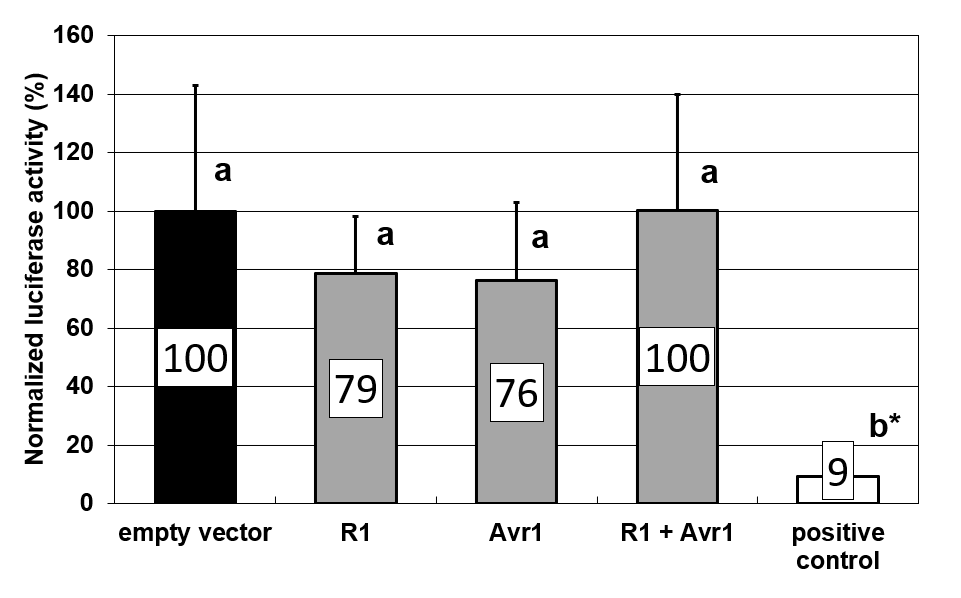

Supplement: Supplementary file 7 — Figure S7 Potato resistance genes R1, Rpi‐blb3 and Rx are not functional in wheat. [file PBI-23-1683-s006.zip › pbi14615-sup-0007-FigureS7a.png]

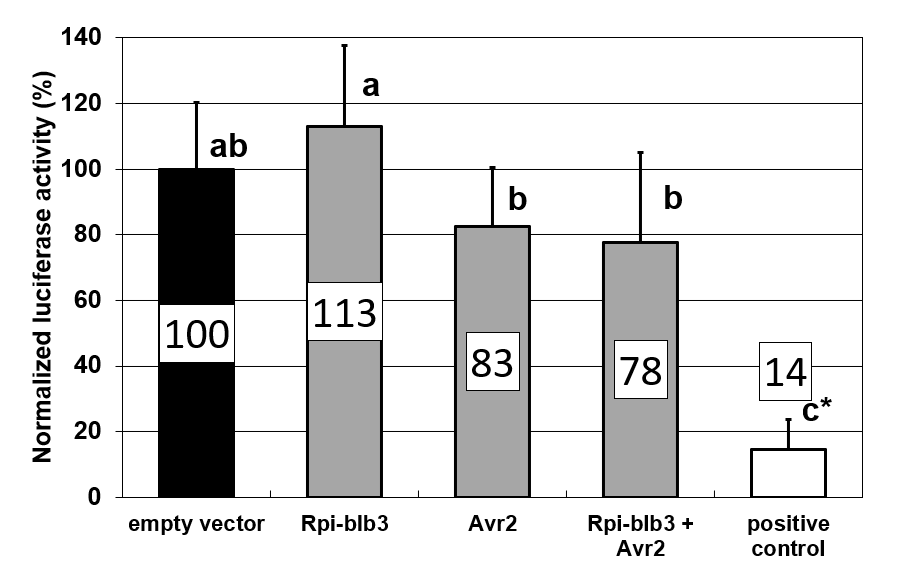

Supplement: Supplementary file 7 — Figure S7 Potato resistance genes R1, Rpi‐blb3 and Rx are not functional in wheat. [file PBI-23-1683-s006.zip › pbi14615-sup-0007-FigureS7b.png]

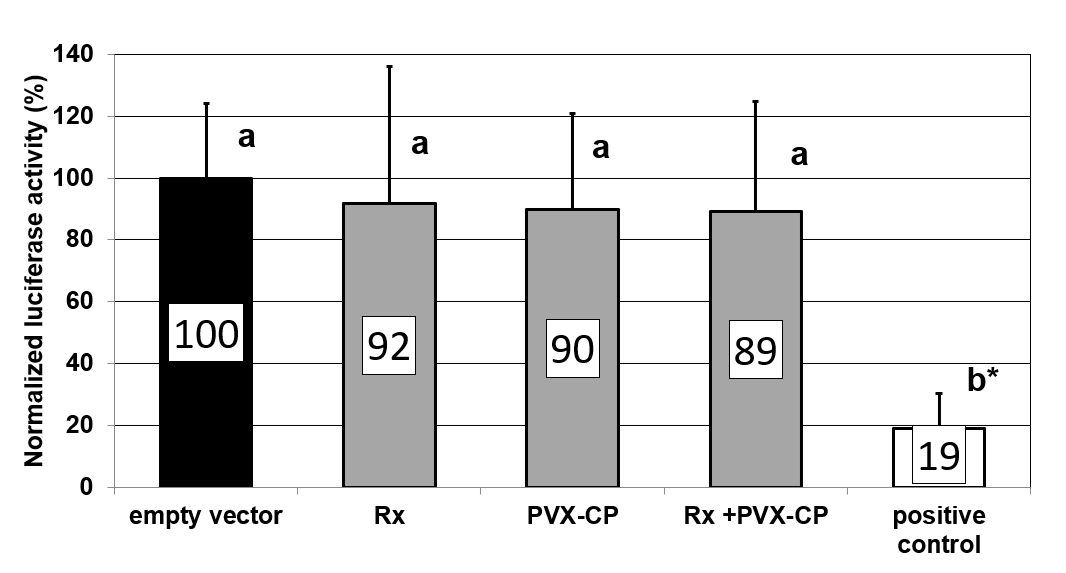

Supplement: Supplementary file 7 — Figure S7 Potato resistance genes R1, Rpi‐blb3 and Rx are not functional in wheat. [file PBI-23-1683-s006.zip › pbi14615-sup-0007-FigureS7c.png]
