# Supplementary material for: Expression of a modified Avr3a gene under the control of a synthetic pathogen‐inducible promoter leads to Phytophthora infestans resistance in potato
Source: Plant Biotechnol J. 2025 Mar 9;23(5):1683–701. doi: 10.1111/pbi.14615 (PMC12018830; doi:10.1111/pbi.14615)
Supplement: Supplementary file 9 — Table S2 Phenotype and late blight resistance of transgenic Baltica lines transformed with 2xS‐4xD‐NpCABEcore::Avr3aKI. [file PBI-23-1683-s007.docx]

**Table S2: Phenotype and late blight resistance of transgenic Baltica lines transformed with 2xS-4xD-NpCABE_core_::Avr3a^KI^**

| **Transgenic lines** | **Phenotype of uninfected plants in the greenhouse** | ***P. infestans* biomass of infected leaves determined by qPCR in comparison to Baltica (DLA assay)** |
| --- | --- | --- |
| Baltica wild type | No phenotype | 100% |
| Baltica-T1 | No phenotype | 100% |
| Baltica-T2 | Small necrotic lesions | 45% |
| Baltica-T3 | Strong dwarf phenotype | Not tested |
| Baltica-T4 | Strong necrotic lesions | 35% |
| Baltica-T5 | Strong necrotic lesions | 85% |
| Baltica-T6 | Necrotic lesions | 90% |
